# Shoreline barriers may amplify coastal groundwater hazards with sea-level rise

**DOI:** 10.1038/s41598-024-66273-w

**Published:** 2024-07-06

**Authors:** Xin Su, Kevin M. Befus, Michelle A. Hummel

**Affiliations:** 1 Department of Geosciences, University of Arkansas, Fayetteville, 72701 AR USA; 2 Center for Applied Earth Science and Engineering Research, University of Memphis, Memphis, 38152 TN USA; 3 Department of Civil Engineering, University of Texas at Arlington, Arlington, 76019 TX USA

**Keywords:** Environmental sciences, Hydrology, Natural hazards, Engineering

## Abstract

Subsurface barriers have been proposed to protect coastal aquifers from sea-level rise induced seawater intrusion, but the potential for groundwater emergence near subsurface barriers remains unknown. Here, we investigated how emergence changes groundwater flow conditions and influences the protective performance of subsurface barriers with sea-level rise. We tested the subterranean consequences of sea-level rise for cutoff walls and subsurface dams with cross-shore groundwater flow and salt transport models, investigating how barrier design, aquifer properties, and hydrological conditions control the potential for emergence, groundwater partitioning at the barrier, and seawater intrusion with sea-level rise. We find that most subsurface infrastructure cannot prevent seawater intrusion and emergence simultaneously. Subsurface dams spanning more than half of the aquifer thickness created emergence hazards and subsequent groundwater partitioning for all scenarios tested. Cutoff walls were less effective at reducing seawater intrusion for all opening sizes but could reduce the emergence potential compared to similarly sized subsurface dams. Our results demonstrate the challenging trade-offs in mitigating the coastal groundwater hazards of seawater intrusion and emergence with sea-level rise, where groundwater flooding inland of protective infrastructure would require combinations of subsurface impoundments and other mitigation techniques, such as pumping or drains.

## Introduction

Globally, over a billion people in low-lying coastal communities face the existential threat of sea-level rise in the coming century^1,2^. Exacerbated by global climate change, sea-level rise has accelerated from 1.4 to 3.6 mm/year from the last century to 2015^3,4^, accentuating the need for understanding climate risks and developing solutions for coastal communities^5^. Low-lying coastal regions are the most prone to flooding even without sea-level rise, often caused by extreme events^6–8^. As such, coastal communities have focused on developing protective infrastructure to reduce flooding, implementing a diverse combination of seawalls, dikes, revetments, and gates^9^. With sea-level rise, these structures may need to be expanded or elevated to maintain their performance.

An often hidden risk to low-lying coastal communities is how the subsurface hydrology responds to protective infrastructure, in addition to marine conditions, sea-level rise, and climate change. It remains poorly quantified how coastal flood protection accounting only for overland flooding may worsen groundwater hazards or require additional expensive solutions, such as long-term pumping like what has been needed to maintain diked lands in the Netherlands^10^. Seawater intrusion, or the inland encroachment underground of saline groundwater laterally into the fresher groundwater lens, is one such groundwater hazard that degrades shallow groundwater quality^11,12^ and is exacerbated by pumping^13^. Even without pumping, sea-level rise causes seawater intrusion by raising the head of saline groundwater at the shore with intrusion occurring laterally into the terrestrial aquifer^14–16^. Storm surge-related flooding can also lead to event-based salinization of unconfined aquifers via surficial seawater infiltrating downward into the terrestrial aquifer^17,18^. Both geological and hydrological factors control aquifer vulnerability to seawater intrusion, which can be grouped into two categories: flux-controlled and topography-limited systems^19–23^. Flux-controlled aquifers maintain a stable seaward hydraulic gradient with sea-level rise, such that seawater intrusion is minimal. In topography-limited systems, a shallow water table near the land surface results in increased potential for the water table to intersect the topography and discharge with rising sea level. The inability of the landward water table to rise unrestricted by topography leads to a reduced hydraulic gradient and seawater intrusion in these systems^19,22^.

**Figure 1:**
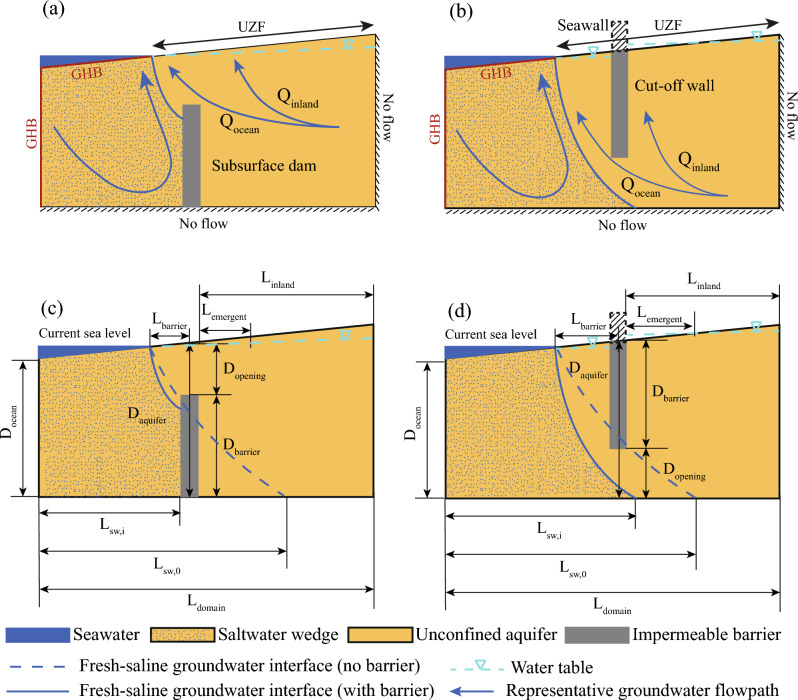
Coastal groundwater flow system conceptual diagram with (**a**) a subsurface dam, and (**b**) a cutoff wall demonstrates dominant groundwater flow pathways. Several geometric variables set the extents of groundwater system for (**c**) the subsurface dam and (**d**) the cutoff wall. A seawall is represented as a dashed feature for the cutoff wall (**b,d**), as a cutoff wall could be an intentional or unintentional foundation to a seawall. *GHB* general head boundary, *UZF* unsaturated zone flow boundary, *Q* groundwater discharge, *D* vertical length scale, *L* horizontal length scale. Definitions of the variables in the figure are further explained in the “Methods”.

To protect the shallow groundwater resources of coastal communities and minimize seawater intrusion, several types of subsurface barriers have been proposed, including physical and hydraulic barriers^24,25^. Physical barriers include subsurface dams, which are constructed upward from an impermeable unit located at the bottom of a shallow aquifer (Fig. 1a), and cutoff walls, which extend downward from the top of an aquifer, either near the water table or land surface, to some depth into the aquifer (Fig. 1b). Both of these subsurface barriers can be constructed with either impermeable (e.g., concrete) or low permeability (e.g., clay slurry) materials^25–27^. Combinations of subsurface dams and cutoff walls can provide additional protection against seawater intrusion^28^. Saltwater intrusion prevention structures have already been built with examples of cutoff walls in China^29,30^ and subsurface dams in Japan^25,31^. Hydraulic subsurface barriers instead use injection wells to reduce intrusion by augmenting the natural hydraulic gradient^32,33^, although this approach has primarily been implemented for deeper confined aquifers^19,34–36^.Therefore, these subsurface barriers could provide additional coastal resilience by protecting freshwater resources against intrusion, and the subsurface infrastructure could be built in coordination with flood protection systems, such as by extending the foundations of seawalls deeper to create a cutoff wall.

Globally, low-lying coastal communities and assets are also threatened by groundwater emergence in unconfined coastal aquifers with projected relative sea-level rise^22,23,37,38^, another potential groundwater hazard that protective coastal infrastructure and climate change could exacerbate beyond seawater intrusion. We hypothesize that physical subsurface barriers could amplify the risk of groundwater emergence and associated hazards. This may occur by restricting coastal flow paths, potentially causing groundwater flooding in poorly drained areas, or reducing infiltration capacity such that flooding due to extreme precipitation worsens in coastal areas^39^. Additionally, these barriers might introduce new groundwater discharge features that sufficiently reduce flow under the barrier to allow more intrusion^40,41^, or lead to new sources of aquifer and soil salinization^42^. Moreover, subsurface barriers alter groundwater flow at the coast and can reduce submarine groundwater discharge^43^. However, most studies on subsurface barriers have focused on protecting against seawater intrusion and have used boundary conditions and assumptions that neglect the potential for water table rise sufficient to cause groundwater emergence with barrier construction and sea-level rise (e.g., prescribed water table elevation or no-flow upper boundaries). Thus, subsurface barriers in unconfined aquifers allowed to have changing water tables through the use of a free surface boundary could lead to unexpected intrusion caused by emergence altering groundwater flow behavior that could be important for effective coastal protective infrastructure.

We develop a simple numerical framework to test how sea-level rise, subsurface barrier construction, and hydrogeologic parameters influence groundwater emergence, seawater intrusion, and groundwater flow partitioning. Unlike previous studies, we solve for the position of the water table as a free surface within the models to quantify the role of emergence and consequent inland discharge on subsurface barrier protective performance. We demonstrate that groundwater emergence causing discharge inland of the barrier is a common hydrologic outcome caused by building subsurface barriers across a wide range of realistic aquifer scenarios. Thus, emergence should be considered when designing infrastructure focused on minimizing coastal flooding and intrusion to avoid creating new hydrologic hazards in coastal areas.

## Results

A total of 3024 groundwater flow and salt transport simulations were run to understand how a range of parameter combinations influenced the modeled behavior (Supplemental Figs. S1–S8). The primary parameters tested included: (1) the recharge ratio, *K*/*r*, defined as the horizontal hydraulic conductivity, *K*, divided by the recharge rate, *r*; (2) the relative opening, $D^*$, defined as the opening thickness above or below the barrier, $D_{opening}$, divided by the thickness of the aquifer, $D_{aquifer}$ (Eq. 1); (3) the relative location of the barrier, $L^*$, defined as the horizontal distance of the barrier from the initial shoreline, $L_{barrier}$, divided by the total domain length, $L_{domain}$ (Eq. 2); and (4) the topographic slope, *S*. Simulations were run for both a free surface condition (UZF) and a prescribed head boundary condition (CHD) at the model’s upper boundary. More details on the range of parameter values tested and the boundary condition implementation are provided in the “Methods”. An additional 3024 spin-up models set the initial conditions for the full simulations, and 48 more models did not include a subsurface barrier to allow a quantitative comparison between the original flow system and the modified system with a barrier.

Three interdependent metrics were used to understand the barrier performance: (1) the inland groundwater emergence ratio, $R_{emergence}$ (Eq. 5), representing the emergence extent relative to the size of the domain; (2) the seawater intrusion reduction ratio, $R_{intrusion}$ (Eq. 3), representing the reduction in intrusion with the barrier relative to no barrier; and (3) the inland groundwater flow ratio, $R_{flow}$ (Eq. 4), representing the ratio of groundwater seepage to the land surface inland of the barrier relative to the total recharge volume. Groundwater emergence inland of a barrier is a prerequisite for the groundwater discharge inland of the barrier to occur, as described by the inland groundwater flow ratio. Conversely, groundwater emergence is not required for changes in the seawater intrusion ratio, but groundwater emergence and the resulting flow partitioning collectively have a strong influence on intrusion. Further development of these metrics is described in the “Methods”. For each metric, we first establish the differences between the free surface (UZF) and prescribed head (CHD) boundary conditions, demonstrating the limitations of previous studies using only the prescribed head formulation. Through this comparison of formulations, we establish that subsurface barriers can lead to groundwater emergence. Next, we analyze how such emergence alters the protective performance of subsurface barriers against seawater intrusion. Finally, we explore how groundwater flow partitioning resulting from emergence can explain the reduced intrusion performance.

### Barrier effects on groundwater emergence

**Figure 2:**
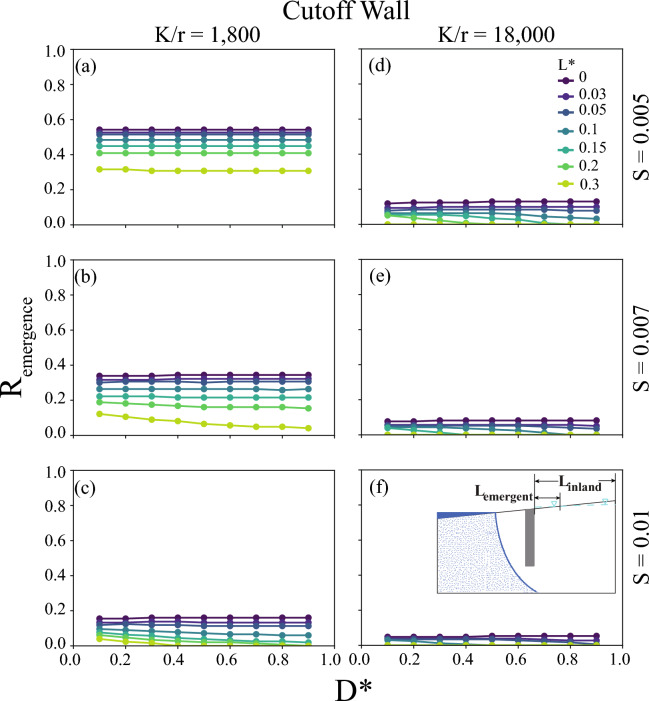
The groundwater emergence ratio, $R_{emergence}$, simulated using the free surface formulation (i.e., UZF) for a cutoff wall. (**a**–**e**) Columns are labeled by the recharge ratio, *K*/*r*, used for the modeling, and the rows are labeled by the topographic slope, *S*. Each point represents two simulations used to calculate $R_{emergence}$, one without a barrier and one with a barrier. Subset diagrams in (**f**) highlight the length scales which make up $R_{emergence}$ calculated with Eq. (5).

**Figure 3:**
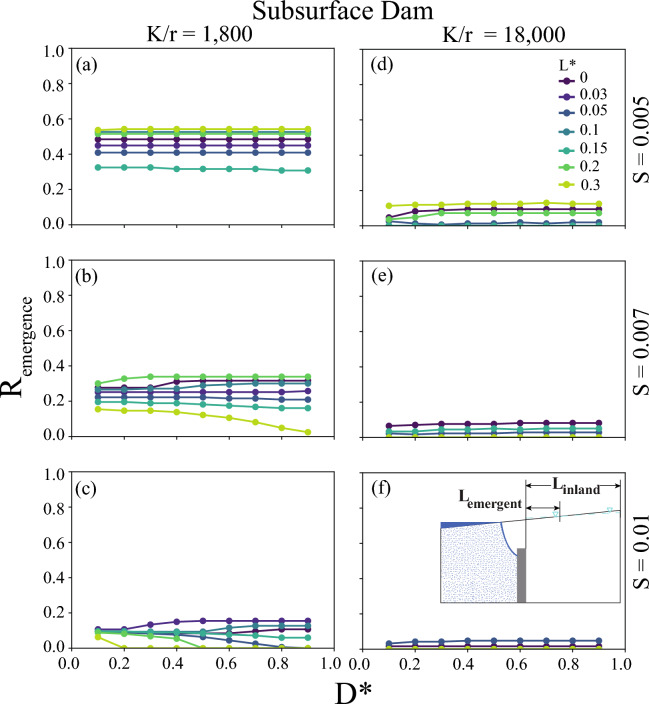
The groundwater emergence ratio, $R_{emergence}$, simulated using the free surface formulation (i.e., UZF) for a subsurface dam. (**a**–**e**) Columns are labeled by the recharge ratio, *K*/*r*, used for the modeling, and the rows are labeled by the topographic slope, *S*. Each point represents two simulations used to calculate $R_{emergence}$, one without a barrier and one with a barrier. Subset diagrams in (**f**) highlight the length scales which make up $R_{emergence}$ calculated with Eq. (5).

Inland groundwater emergence can be a direct consequence of the groundwater flow being diverted upward to the land surface inland of the barrier, as has been studied previously for areas without barriers^23,38^. The portion of the model domain inland of the barrier with groundwater emergence, quantified by $R_{emergence}$ (Eq. 5), varied from 0 (i.e., no groundwater emergence) to 1 in our simulations (Fig. 2 and 3). Simulations with $K/r \le 180$, representing very topographically-limited aquifer conditions, resulted in groundwater emergence nearly everywhere inland of the barriers ($R_{emergence}\ge 0.8$), including for scenarios without barriers for most parameter combinations (Supplemental Figs. S9 and S10). Across all cutoff wall scenarios, the most emergence occurred with $L=0$, as the barriers were located at the present-day coastline and at the lowest terrestrial discharge area for the groundwater flow system (Fig. 2). The horizontal position of the barriers, $L^*$, was the primary control for $R_{emergence}$ for each *K*/*r* for both cutoff walls and subsurface dams, although some of this relationship is caused by the shortening of the upland extent used in calculating $R_{emergence}$. Moving the barrier inland increased its influence on the emergence ($R_{emergence}$), showing greater control of the relative barrier height (*D*) on groundwater flow paths. Conversely, altering the aquifer opening above or below the barrier through *D* generally resulted in minimal changes or decreases in $R_{emergence}$, suggesting limited influence of the barrier on groundwater levels. Scenarios with changes in $R_{emergence}$ with changes in the barrier opening, $D^*$, are expected to arise for barriers that alter the groundwater flow relative to natural conditions. As $R_{emergence}$ was influenced by the horizontal resolution of the model and the relatively simple representation of the land surface topography, these results could not resolve smaller scale changes in emergence. While the extent of emergence did not change substantially across these simulations, the existence and expansion of groundwater emergence led to less seawater intrusion protection (i.e., lower $R_{intrusion}$) by increasing the groundwater flow inland of the barriers (i.e., higher $R_{flow}$), as discussed in the following sections.

### Barrier effects on intrusion protection

With the $R_{emergence}$ analysis demonstrating the ubiquity of groundwater emergence inland of subsurface barriers, we tested the effectiveness of the barriers at blocking seawater intrusion using $R_{intrusion}$ (Eq. 3). Unlike previous studies that specified only constantly sloping water table position, a “prescribed head model”, we solved for the water table position to allow for water table rise with “free surface models”. Since intrusion was the primary focus of previous investigations of subsurface barriers using only prescribed water tables^26,44^, we analyze how allowing groundwater emergence using the free surface formulation (i.e., UZF boundary) altered and generally reduced the amount of intrusion protection relative to using a prescribed water table (i.e., CHD boundary). Then, as the free surface condition is the more realistic of the model formulations, we analyze the behavior of $R_{intrusion}$ in the free surface models across a wide range of scenarios.

**Figure 4:**
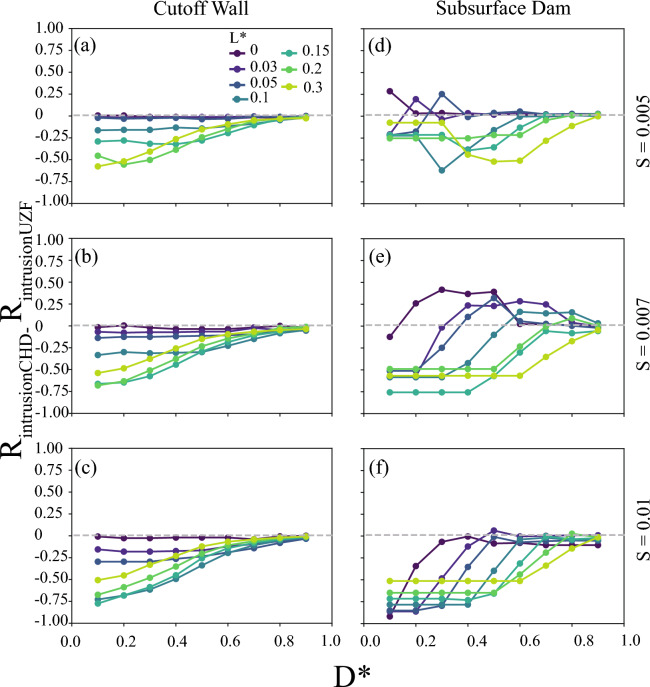
The difference between the seawater intrusion reduction ratio ($R_{intrusion}$) from the prescribed head (CHD) to the free surface (UZF) formulations for scenarios varying barrier opening ($D^*$) and barrier location ($L^*$) for certain land surface slopes (*S*), comparison with (**a–d**) a cutoff wall implementation and (**e–h**) a subsurface dam implementation with high recharge ratios, $K/R = 18,000$, demonstrating the pervasive and parameter-dependent importance of allowing seepage with a free surface formulation to realistically compute the amount of saltwater intrusion reduction. The rows are labeled by the topographic slope, *S*. Each point represents two simulations in calculating $R_{intrusion}$ for each implementation.

We found substantial differences in the intrusion protection, $R_{intrusion}$, between models solving for the water table position relative to the prescribed head models, as used in previous studies (Fig. 4; Supplemental Figs. S11 and S12). Interestingly, the mechanism for this intrusion protection difference depended on the sign of the difference. For example, an intrusion protection difference greater than 0 by the prescribed head model occurred when the prescribed head model simulated more relative intrusion protection by not allowing emergence to reduce the flow in the aquifer at the barrier than the free surface model for a given barrier (e.g., more intrusion in the free surface model, $L_{SW,i_{CHD}} < L_{SW,i_{UZF}}$) (Fig. 4). Conversely, a difference in the intrusion protection between the model types of less than 0 occurred when the original position of the saline wedge was further inland for the free surface model than the prescribed head model (i.e.,$L_{SW,0_{UZF}} > L_{SW,0_{CHD}}$), and the influence of the barrier on the intrusion mattered less in the prescribed head model given the starting position of the interface. Thus, the representation, shape, and position of the water table between these simulation types substantially influenced the how the barriers altered the shape and position of the saline groundwater wedge. Overall, the result of allowing groundwater emergence by using the free surface UZF formulation was that both subsurface barriers provided less intrusion benefits in the aquifer, since conditions where barriers provided more intrusion benefits with the free surface formulation resulted in more extensive intrusion both with and without a barrier compared to the prescribed head formulation. However, the UZF formulation further indicated that subsurface barriers can provide substantial reductions in seawater intrusion for aquifers with the potential for groundwater emergence (i.e., topography-limited). We investigate these effects further in the following section, focusing specifically on how these boundary conditions affected the groundwater flow partitioning that led to these differences in intrusion protection.

**Figure 5:**
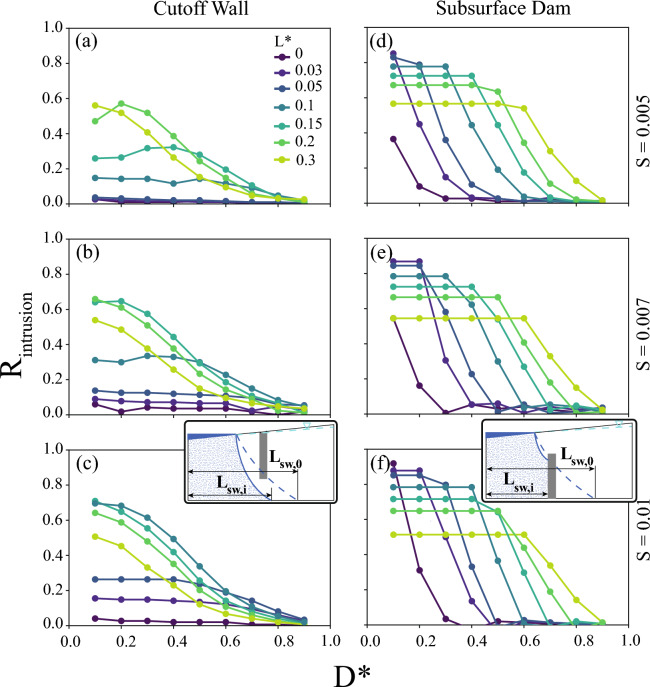
Seawater intrusion reduction ratio ($R_{intrusion}$) for the free surface model (i.e., UZF) scenarios varying barrier opening ($D^*$) and barrier location ($L^*$) for certain land surface slopes (*S*), comparison with (**a–c**) a cutoff wall implementation and (**d–f**) a subsurface dam implementation with high recharge ratios, $K/R = 18,000$. The rows are labeled by the topographic slope, *S*. Each point represents two simulations in calculating $R_{intrusion}$ for each implementation. Subset diagrams in (**c,f**) highlight the intrusion length scales which make up $R_{intrusion}$ (Eq. 3).

Since the free surface models showed different and generally less intrusion protection relative to the prescribed water tables, we tested how the model scenarios influenced the intrusion protection specifically in the free surface models. Results for the cutoff wall demonstrated overall lower $R_{intrusion}$ than subsurface dams, matching findings from previous prescribed water table studies^27,45^ (Fig. 5). For both barriers, increasingly flux-controlled flow systems (i.e., increasing *K*/*r* ratio in Supplemental Figs. S13 and S14) led to more intrusion protection, with the largest barriers relative to the aquifer thickness (low $D^*$) and closest to the coast (low $L^*$) also offering more protection relative to smaller barriers. Cutoff walls and subsurface dams provided up to a 71% and 91% reduction, respectively, in seawater intrusion for the most flux-controlled case (i.e., $K/r=18,000$). The barriers were relatively less effective (i.e., lower $R_{intrusion}$) for flatter topographic settings and were more effective for steeper topographies (e.g., Fig. 5 a–c, and d–f). Further analysis of the $R_{intrusion}$ results is available in the “Evaluation of indicator results” section in the Supplemental Information.

### Barrier effects on groundwater flow partitioning

**Figure 6:**
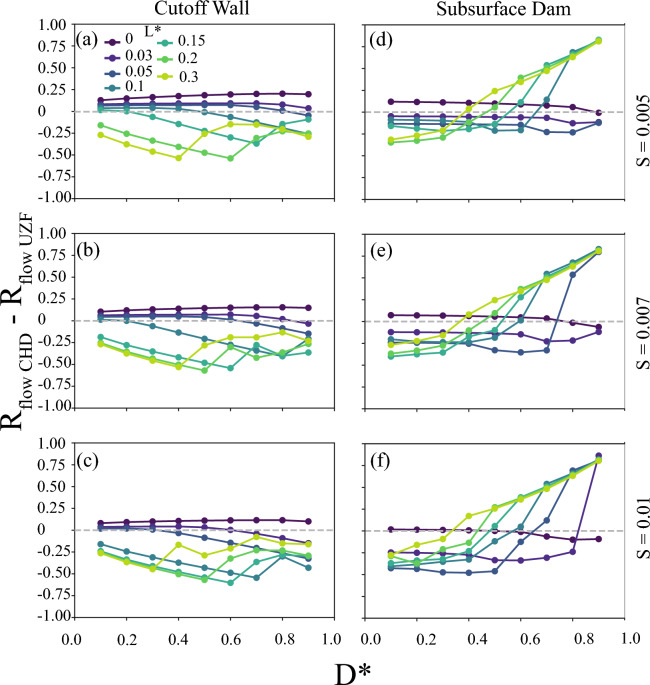
Difference of the inland flow ratio ($R_{flow}$) from the prescribed head (CHD) to the free surface (UZF) formulations with (**a–d**) a cutoff wall implementation and (**e–h**) a subsurface dam implementation with high recharge ratios, $K/R = 18,000$, demonstrating the pervasive and parameter-dependent importance of allowing seepage with a free surface formulation to alter flow partitioning. The rows are labeled by the topographic slope, *S*.

Finally, we explored the influence of groundwater emergence on the increase of the ratio of inland groundwater flow ($R_{flow}$) through the free surface models. We compared the groundwater flow partitioning of the free surface and prescribed head approaches to understand how groundwater emergence limited the saltwater intrusion benefits in the free surface models, revealing major differences (Fig. 6 and Supplemental Figs. S15 and S16). A positive difference of $R_{flow}$ indicated proportionally more discharge inland of the barrier for the prescribed head simulation compared to the free surface simulation. It is important to note that the total recharge, *r*, used to calculate $R_{flow}$ for each implementation was not constant between models, as both *r* or $Q_{inland}$ could change the $R_{flow}$ in this analysis depending on the type of boundary condition.

For cutoff walls blocking much of the aquifer (low $D^*$) and close to shore (low $L^*$), the prescribed head implementation predicted higher proportions of discharge to recharge inland of the barrier for almost all of the $R_{flow}$ values (Fig. 6a–c and Supplemental Fig. S15). With $K/r=18,000$, prescribed head models of cutoff walls continued this trend for most $D^*$ configurations with $R_{flow}$ reaching up to 24$\%$ for $L^*<0.05$. Similarly, this transition from prescribed head predicting larger $R_{flow}$ to free surface models predicting larger $R_{flow}$ occurred with increasing $D^*$ for $K/r>=180$ scenarios (Supplemental Fig. S14d–l). Steeper topography in the cutoff wall models resulted in either less of a positive difference between the $R_{flow}$ values or a more negative difference, resulting from more topographic discharge feedback in the free surface models.

Comparison of the free surface-based subsurface dam simulations with the simplified prescribed head models again revealed major effects on $R_{flow}$ (Fig. 6d–f and Supplemental Fig. S18). For $K/r \le 180$, most conditions ($L^* > 0.03$) led to lower $R_{flow}$ in the prescribed head implementation by up to  50$\%$ than the free surface models. Furthermore, free surface-based subsurface dam models predicted higher $R_{flow}$ of up to 62$\%$ with increasing $L^*$ (Fig. 6d–f and Supplemental Fig. S15 j–l). Substantial higher $R_{flow}$ in the prescribed head models occurred for $L^*<0.03$, indicating the large $R_{flow}$ dissimilarity between the boundary conditions with barriers very near the coastline. For the lower $L^*$ scenarios, the simulated $R_{flow}$ difference decreased from larger $R_{flow}$ with prescribed head with increasing $D^*$ to very little to no difference (e.g., Supplemental Fig. S16d), highlighting the importance of how the boundary conditions at the surface were affected by changing aquifer parameterization. Larger values of $L^*$ with barriers farther inland tended to increase the $R_{flow}$ from the prescribed head implementation slightly with unblocked portion of the aquifer, $D^*$, for low *K*/*r* scenarios, and the $R_{flow}$ increase became more substantial with higher *K*/*r* (e.g., Fig. 6d–f and Supplemental Fig. S16b, e, h, k).

In addition, comparing how the upper boundary conditions represented topography-limited or flux-controlled conditions in the aquifer provided additional context for evaluating the effect of the barriers on flow partitioning. Larger under predictions of $R_{flow}$ by the prescribed head models occurred for flux-controlled conditions (i.e., high *K*/*r*) with cutoff walls further inland (Supplemental Fig. S14j–l), restricting the extent and fluxes from an inland seepage face relative to the free surface modeling results. Both implementations for topography-limited conditions for both barrier types were more similar for the low *K*/*r* simulations than for the more flux-controlled, high *K*/*r* simulations. Importantly, the influence of the overtopping of the subsurface dam by the saltwater wedge on the free surface model $R_{flow}$ did not occur to the same degree for the prescribed head model $R_{flow}$ results, leading to the larger $R_{flow}$ in the prescribed head models most notably for the high *K*/*r* and more inland (i.e., high $L^*$) subsurface dam simulations.

**Figure 7:**
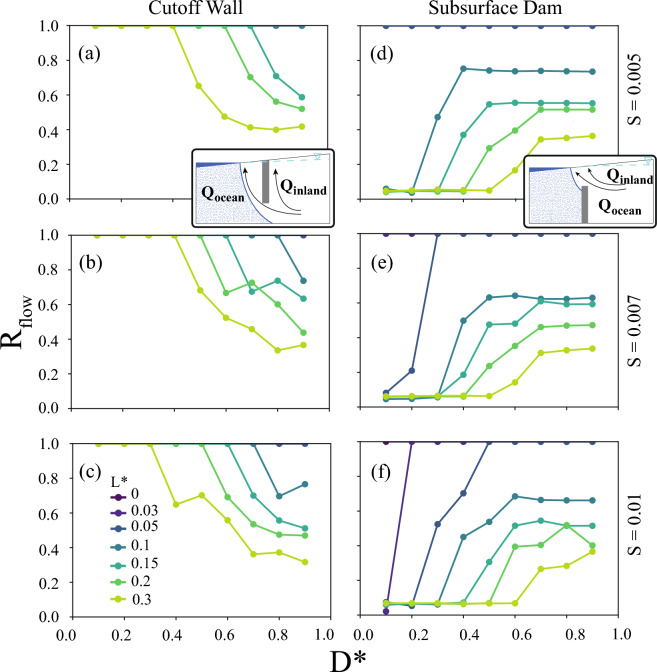
Inland flow ratio ($R_{flow}$) for the free surface formulation (i.e., UZF) with (**a–d**) a cutoff wall implementation and (**e–h**) a subsurface dam implementation with high recharge ratios, $K/R = 18,000$. The rows are labeled by the topographic slope, *S*. Subset diagrams in (**a,d**) highlight the discharges which make up $R_{flow}$ (Eq. 4).

When using the free surface model formulation that allowed groundwater emergence, the $R_{flow}$ results for the cutoff wall simulations demonstrated the ubiquity of groundwater discharge to the surface inland of the barrier across all scenarios tested (Fig. 7 and Supplemental Figs. S17 and S18). None of the cutoff wall scenarios tested in this analysis resulted in $R_{flow} = 0$, where the minimum value reached was 0.035 ($K/r=1800$ at large $D^*$ and $L^*$). Increasing $L^*$ led to decreased $R_{flow}$, as the barrier was installed farther from the shore and higher up slope. Since the cutoff wall extends from the land surface downward, moving the barrier inland also raised the location of the barrier to a thicker area of the aquifer. Cutoff wall $R_{flow}$ varied across its full range with the parameter space tested for each *K*/*r*. Only for cutoff walls close to the coast (i.e., low $L^*$) and more flux-controlled conditions (i.e., relatively high *K*/*r*) did $R_{flow}$ remain constant at a value of 1, indicating all surficial groundwater discharge occurred inland of the barrier due primarily to the saline groundwater wedge extending inland of the cutoff wall (Supplemental Fig. S17). Another flow partitioning effect of the cutoff wall was that increasing $D^*$ lowered $R_{flow}$, as this shallower cutoff wall allowed more groundwater to flow under the barrier.

The $R_{flow}$ results for the subsurface dam in the free surface models allowing emergence (in Fig. 7d–f and Supplemental Fig. S18) were also sensitive to the parameters spanning real-world conditions, although the subsurface dam models resulted in fewer cases with substantial inland discharge than the cutoff walls (i.e., $R_{flow}$ less frequently equal to 1) (Supplemental Fig. S18). At low *K*/*r* (Supplemental Fig. S18a–c), all but the most nearshore (i.e., lowest $L^*$) scenarios were sensitive to groundwater discharging inland of the subsurface dam with increasing discharge inland (i.e., increasing $R_{flow}$) as the aquifer opening decreased (i.e., lower $D^*$). The saline groundwater wedge intruded inland of the subsurface dam for the lowest $L^*$ scenarios (Supplemental Fig. S18), focusing groundwater discharge inland of the barrier under most scenarios. An inland groundwater discharge minimum occurred in several $K/r >= 180$ scenarios (Supplemental Fig. S18d–l), where increasing the aquifer opening (i.e., increasing $D^*$) reduced the inland discharge until the saline groundwater wedge could over top the subsurface dam. Inland groundwater discharge for overtopped subsurface dams was then affected by the position of the saline wedge and the barrier within the flow system, and the $R_{flow}$ either increased or stabilized.

## Discussion

Numerous studies have advanced the understanding of how coastal barriers can prevent saltwater intrusion with physical sandbox experiments and numerical models^24–26,33,43,46–48^. However, previous work overlooked the interaction of surface water and groundwater upstream of the barriers by applying a prescribed head inland boundary condition or by only considering conditions where the water table would not emerge inland of the barrier. Our simulations tested conditions beyond these limitations by implementing models across a wide range of *K*/*r* (i.e., hydrogeologic and climatic conditions) and using both the prescribed head and free surface boundary conditions along the land surface. Our analysis indicated that using a static water table slope with the prescribed head boundary resulted in substantial differences in flow partitioning (i.e., $R_{flow}$) and saltwater intrusion (i.e., $R_{intrusion}$) relative to the more adaptive free surface simulations. Our results also quantified that such simplification of the water table and groundwater flow partitioning in previous analyses would result in 5% and up to nearly 100% misrepresentation of the extent of saltwater intrusion prevented by subsurface barriers based on the difference between the two implementations (Supplemental Figs. S11, S12, S14, and S16). These differences were most substantial for scenarios that were flux-controlled (i.e., high recharge ratio, *K*/*r*) (e.g. Figs. 4, and 6). This was not the expected result, since topography-limited conditions would be expected to create the most overall flow partitioning resulting in groundwater discharge inland of the barrier, as the water table is more likely to intersect and drain from the land surface^23^. Instead, we believe the larger differences between the model types for the flux-controlled conditions were a result of the free surface models solving for a higher water table inland of the barrier caused by the barrier deflecting some portion of groundwater flow inland (i.e., as quantified by $R_{flow}$). We interpreted the overall similarity between the two model implementations for topography-limited scenarios to be caused by both boundary conditions allowing similar amounts of groundwater discharge inland of the barrier. Thus, the importance of considering a feedback between groundwater discharge and water table position would be most important when designing subsurface barriers for flux-controlled aquifers with the potential for the barrier or changing hydrologic conditions to create new groundwater emergence inland of the barrier.

The simulation results indicated that the intrusion benefit of barriers ($R_{intrusion}$) generally decreased with increasing aquifer openings ($D^*$) for most models at a fixed distance from shore ($L^*$) due to the decrease in barrier height. Interestingly, our results also showed that the minimum effective subsurface dam for an identical aquifer depended on the location of the barrier placement, $L^*$. We found that subsurface dams displayed high and consistent intrusion benefits (i.e., $R_{intrusion}$) at small aquifer openings (i.e., low $D^*$), which rapidly decayed to no intrusion protection benefit ($R_{intrusion}=0$) beyond a threshold where the top of the subsurface dam intersected the fresh-saline interface. This threshold was determined by the height of the dam and its placement, and subsurface dams were too short to prevent seawater intrusion over the top of the dam for openings larger than this threshold. We also found that subsurface dams were more sensitive to the aquifer opening size ($D^*$) than cutoff walls.

The finding that all cutoff walls led to some groundwater discharge inland of the barrier (see “Barrier effects on groundwater emergence”) assumed that the barrier extends at least to the water table. Under some construction conditions in more permeable and/or arid environments, shallower foundations of coastal barriers, such as seawalls, might not intersect the water table nor cause the groundwater partitioning our analysis found. However, in such settings with a deep water table, the design of the cutoff wall would not limit saltwater intrusion, as all such barriers would have to at least intersect the water table to influence lateral groundwater flow. Instead, the foundations and subsurface construction for seawalls or similar preventative coastal infrastructure could act as cutoff walls for groundwater flow and initiate unexpected groundwater emergence.

In addition to preventing saltwater intrusion, subsurface barriers that change groundwater flow and water levels could instigate or initialize other chemical hazards^49^. For example, the design and construction of subsurface barriers could aim to limit the transport of various chemical species or contaminants in submarine groundwater discharge^27,50^. However, our study showed that limiting such discharge by building a subsurface barrier could also increase groundwater discharge inland of the barrier, shortening reactive flowpath lengths and potentially creating new pathways for contamination to reach the land surface via groundwater emergence. Further, our results indicated that building a subsurface barrier could raise the water table, which even without emergence could mobilize or remobilize vadose zone contamination^51^. Water table rise associated with subsurface barrier construction could also enhance liquefaction risk^52^ if no additional dewatering features are built^53^. However, many liquefaction mitigation methods reduce shallow sediment permeability (i.e., solidification or densification) and could act as cutoff walls for groundwater flow^54,55^. Therefore, our study provides new insights into how constructing subsurface barriers today to protect against long-term sea-level rise or present-day liquefaction hazards may increase fresh groundwater discharge inland of subsurface barriers, expand areas with groundwater emergence, and potentially lead to inland groundwater-transported contaminant concerns.

**Figure 8:**
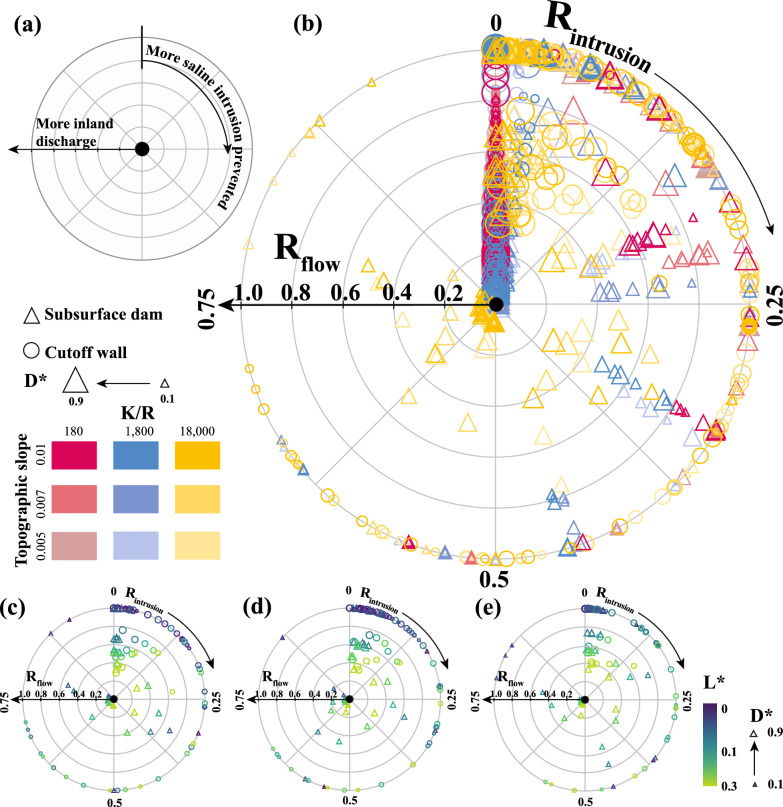
Multi-hazard barrier effectiveness trade-offs between the saltwater intrusion reduction ratio ($R_{intrusion}$) calculated by Eq. (3) and the inland groundwater flow ratio ($R_{flow}$) calculated by Eq. (4) for cutoff walls and subsurface dams with the free surface models. (**a**) Overview of the axes of the trade-off wheel. (**b**) Composite trade-off wheel for all model results for both cutoff walls and subsurface dams with each point representing two simulations used to calculate each ratio, one without the barrier and one with the barrier. (**c–e**) Trade-off wheels for $K/r = 18,000$ with *S*= 0.005, 0.007, and 0.01 respectively. A table of individual model results are available in the supporting data set (see Supporting Fig. S18).

As described in the “Barrier effects on intrusion protection”, the subsurface barriers considered in our analysis could reduce seawater intrusion for a wide range of conditions. However, both barriers also triggered groundwater emergence by increasing $Q_{inland}$, which to our knowledge is a hydrologic response that has never been investigated. To further understand the apparent trade-off between intrusion protection and groundwater flow partitioning, we introduce the “coastal groundwater protection and multi-hazard trade-off wheel” (Fig. 8 and Supplemental Fig. S19) to integrate both $R_{intrusion}$ and $R_{flow}$ results into a single framework. For most coastal settings, the optimal barrier effectiveness would likely be for construction parameters leading to the largest $R_{intrusion}$ and the smallest $R_{flow}$, moving as far in the clockwise direction around the wheel and as close to the center as physically possible. Unfortunately for maximizing such effectiveness, all free surface simulations for cutoff walls remained in the first three quadrants of the wheel in a clockwise direction (i.e., $R_{intrusion} < 0.75$), with a maximum $R_{intrusion}$ of 0.71 and $R_{flow}=1$ (Fig. 8d). Only 23 subsurface dam scenarios extended beyond $R_{intrusion} > 0.75$ (Fig. 8e). For $K/r\le 1,800$, 18% of subsurface dam scenarios extended beyond $R_{intrusion} > 0.25$ (i.e., beyond first quadrant), while more than 80% of the tested scenarios extended beyond the first octant ($R_{intrusion} > 0.125$). For these low *K*/*r* scenarios, both barriers primarily fell along the $R_{flow}$ axis with $R_{intrusion} ~= 0$, highlighting the challenge of constructing a subsurface barrier and not causing inland groundwater discharge and emergence for these hydrogeologic conditions. Thus, the extent to which a shallow coastal aquifer is or becomes topography-limited with sea-level rise, climate change, or human activities could shift how a barrier could affect intrusion, groundwater flow partitioning, and emergence (i.e., changing the position on the multi-hazard trade-off wheel). Overall, subsurface dams provided more intrusion protection with less emergence potential over a larger parameter range, although the hydrologic setting of any barrier will evolve with sea-level rise.

We developed this multi-hazard barrier effectiveness framework to allow first-order planning and management decisions for intrusion protection projects. Coastal surficial flooding protection projects could also use these results when designing subterranean components (e.g., substructures or foundations). These model results demonstrate that fully blocking seawater intrusion with only physical barriers is not possible for the extensive parameter combinations we tested, even with the relatively small amount of sea-level rise we considered (i.e., 36 cm). Beyond finding no models achieving $R_{intrusion}=1$, these simulations also showed the linked and ubiquitous emergent groundwater hazard associated with building physical subsurface barriers. The multi-hazard trade-off wheel can provide an initial framework for developing project values and targets related to how much intrusion would be permissible at a site relative to the allowable groundwater emergence, although additional consideration of pumping and site-specific simulations may be needed for more precise results.

Furthermore, there are additional nuances to the groundwater emergence and seawater intrusion trade-offs when building subsurface barriers. For example, groundwater emergence could be a beneficial outcome for such construction if the $Q_{inland}$ could support wetland restoration, migration, or development, where simulations resulting in higher $R_{flow}$ would indicate more freshwater discharge to support wetland development inland of a barrier. These wetlands could be fresh to saline depending on the position of the fresh-saline interface, providing a wetland migration corridor inland of grey infrastructure that could otherwise limit migration potential. In more arid regions, increases in $Q_{inland}$ and groundwater emergence could lead to salinization of shallow groundwater due to concentrating salts via evaporation^56–59^. Pumping as a solution to increasing $Q_{inland}$ creates additional challenges, as such pumping could reduce groundwater emergence at the cost of allowing more intrusion. Thus, additional research is needed to understand how the emergence challenge we found in this study can be mediated without exacerbating subsurface salinity intrusion, such as with hydraulic barriers. By representing groundwater emergence and surficial discharge caused by a subsurface barrier, our research provides a more complete understanding of the effects barriers have on the coastal groundwater flow system, which can help inform the design and implementation of effective coastal barriers to specifically prevent saltwater intrusion or provide a more holistic hydrologic and hydrogeologic perspective for protective infrastructure impacting subsurface flow conditions.

Finally, there is increasing interest in natural and nature-based coastal infrastructure as a means of protecting coastal communities and providing additional ecosystem services. This may lead to reduced investment in traditional grey infrastructure, which often relies on concrete and other engineered materials^60^. Our study focused on low permeability barrier constructions with a range of barrier openings and locations. However, combining a low permeability cutoff wall with a semi-permeable opening could further reduce saltwater intrusion and limit nitrate contamination^27^. Building on this work, future research could explore other permeability alterations of both the barrier and opening. While no study has yet classified subsurface barriers on the green-to-grey infrastructure continuum^60^, the substantial disturbance required to construct a subsurface barrier and the use of concrete or other engineered materials for the low permeability media suggest that these barriers are more closely related to grey infrastructure^61^. However, the combination of low and semi-permeable barriers may provide a more nature-based solution with greater benefits for both humans and the environment. Further research is needed to fully assess the potential of these approaches and their place within the spectrum of green-to-grey infrastructure options.

In our analysis, we tested how subsurface barriers, specifically cutoff walls and subsurface dams, perform in blocking seawater intrusion across globally representative ranges of hydrogeologic and construction parameters with a moderate amount of sea-level rise. Unlike previous studies of subsurface barriers, our simulations solve for the position of the water table and focus on quantifying the feedback between groundwater emergence and groundwater flow partitioning around the barrier collectively altering the protective effectiveness of the barrier against seawater intrusion. Our simulations showed that solving for the water table while allowing rejected recharge and groundwater discharge inland of the barrier (i.e., free surface implementation) led to substantially different results than more simplified models with a prescribed water table (i.e., prescribed head implementation). The prescribed head results indicated more protection against seawater intrusion (higher $R_{intrusion}$) relative to the more realistic free surface models by up 58%, while more overall intrusion in the free surface models for higher *K*/*R* settings allowed for more protection than the prescribed head models by up to 81%. The cause of these differences is related to groundwater flow partitioning around the barrier with the proportion of inland discharge ($R_{flow}$) differing by up to 94% between the prescribed head and free surface model implementations. Differences between the two model implementations occurred across changes in hydrogeologic and construction parameterizations. These results demonstrated the importance of simulating the complete response of the water table to sea-level rise including groundwater emergence, especially for flux-controlled systems that are or eventually become locally topography-limited with the construction of a subsurface barrier. A primary objective of physical subsurface barrier research has been to evaluate their effectiveness in preventing saltwater intrusion, and the results of our simulations indicated that groundwater flow partitioning inland of the barrier led to substantial reductions in intrusion protection relative to previous studies. Similar to previous work, our free surface models also confirmed the effectiveness of subsurface barrier construction in preventing saltwater intrusion after long-term sea-level rise by up to 71% for cutoff walls and 92% for subsurface dams when compared to cases where no barriers were present.

Importantly, we find that no models led to optimal conditions, where the subsurface barrier could block seawater intrusion while causing no groundwater emergence hazard. Instead, subsurface dams were found to provide the most protection from intrusion but with most conditions leading to excessive groundwater discharge inland of the barrier. Cutoff walls were less effective at blocking seawater intrusion and also led to more groundwater discharge inland of the barrier for equivalent subsurface dam scenarios. As our study showed, the partitioning of inland groundwater by subsurface barriers is a crucial factor in determining the emergence of groundwater and the effectiveness of barriers at blocking saltwater intrusion. This highlights the importance of careful model- and field-based investigations before designing and constructing subsurface barriers, taking into account local conditions such as sea-level rise rates, hydroclimatology, nearshore topography, and hydrogeological properties. Moreover, the potential benefits and drawbacks of groundwater emergence should also be considered when designing and implementing subsurface barriers. Pumping as a solution to mitigate inland freshwater discharge could also exacerbate saltwater intrusion. Therefore, it is crucial to explore alternative methods, such as hydraulic barriers, to address these challenges.

Our study addresses the complexity of managing coastal groundwater resources and highlights the need for comprehensive, site-specific approaches to coastal infrastructure planning that consider both the short- and long-term implementations of protective strategies. The development of the “coastal groundwater protection and multi-hazard trade-off wheel” offers guidance for identifying and balancing intrusion protection and groundwater emergence hazards in coastal management and planning decisions. Future research could further test subsurface barrier designs that can maximize their protective services while taking advantage of groundwater flow partitioning to support coastal water resources and ecosystem resilience.

## Methods

A transient, density-dependent groundwater flow model was developed using MODFLOW 6^62^ to simulate two-dimensional flow and seawater intrusion in a coastal unconfined aquifer with a subsurface barrier. We conceptualize the coastal unconfined groundwater flow system as a two-dimensional, cross-shore flow system comprised of a single homogeneous and isotropic geologic unit overlying a flat, impermeable geologic unit (Fig. 1). Thus, groundwater flow is perpendicular to the coastline. The inland edge of the model is a vertical no-flow boundary representing a groundwater divide. Seaward, the model is truncated offshore with both a prescribed head and salinity boundary in the aquifer that also spans the horizontal inundated marine extent of the seabed. The top model boundary receives recharge and allows discharge such that the elevation of the water table is simulated in the model and not prescribed for the free surface implementation. For the prescribed head implementation, a water table slope is set to the topographic slope. Thus, groundwater recharges along the upper boundary of both model implementations and is either saline or fresh depending on the position of sea level.

Coastal groundwater flow with subsurface barriers was tested within a normalized framework to represent the large range of coastal groundwater systems globally (Fig. 1c,d). Domain dimensions were normalized by the thickness of the aquifer at the barrier, $D_{aquifer}$, which was the sum of the height of the barrier, $D_{barrier}$, and remaining aquifer thickness open to flow, $D_{opening}$. The topographic slope, *S*, was used to translate from the vertical to the horizontal dimension. The recharge ratio, *K*/*r*, defined by the horizontal hydraulic conductivity, *K*, divided by the recharge rate, *r*, was implemented in the models using a fixed *K *= 1.0 m/day with changing recharge rates. *K*/*r* values were selected to span real coastal aquifers, set to 18, 180, 1800, and 18,000^63^.

### Model development

The variable density groundwater flow and dissolved salt transport model consisted of 20 layers each with 200 columns and 1 row to create a structured grid over the two-dimensional cross-section domain. Each layer was 2.5 m thick, and each column was 5 m wide. Two fixed terms for model domain structure were aquifer length, $L_{domain}= 1000$ m, and aquifer thickness at the lowest model elevation, $D_{ocean}= 50$ m. We set the upper model boundary with a constant land surface slope (*S*) to define the surface topography with slopes of 0.005, 0.007, and 0.01, spanning a majority of coastal topographic slopes globally^63^.

Two boundary conditions were tested on the model upper boundary in areas above sea level to create the “free surface” and “prescribed head” implementations. For the free surface implementation, the Unsaturated Zone Flow (UZF) package was used to supply recharge when groundwater heads were below the topography and allow discharge while rejecting any infiltration when heads were at or above the cell elevation^64^. Thus, we used the UZF package to solve for the nonlinear position of the water table dependent on the hydrogeologic setting and net fluxes rather than enforcing an inland prescribed head condition either at the model boundary or to represent the water table position^64,65^. This flexible representation of the water table allows this study to more comprehensively represent the hydrologic setting with subsurface barriers than previous studies^19,22,24,25,27,66,67^. To further quantify the added benefit the UZF boundary provided to the free surface simulations, we also ran prescribed head models using a prescribed water table head equal to the topographic slope with the Time-variant Specified-head (CHD) package. For the free surface models, the model recharge was assigned with a constant infiltration rate (i.e., portion of precipitation entering the subsurface and crossing the water table as potential recharge) with a freshwater concentration of $C_f$ = 0 g/L in the UZF package, with discharging cells rejecting all recharge. The vertical UZF surface depression depths, which represented subgrid-scale topographic variability that could affect drainage, were assigned using the topographic slope multiplied by the horizontal cell width for each cell^62^. Specific yield was kept constant at 0.2 across models, and the residual water content was set to 0.1.

The MODFLOW 6 UZF package was used to simulate unsaturated flow processes in the vadose zone, which includes the region above the water table. However, it does not directly implement an equation of variably-saturated porewater flow (e.g., Richards equation). Instead, the UZF package employs simplified soil property functions, such as the Brooks-Corey model, to approximate unsaturated flow behavior. Regarding the calculation of the water table in our analysis, MODFLOW 6 did not directly use the UZF package for this purpose. The water table position was determined as part of the overall groundwater flow simulation as a dynamic result of the interaction between recharge, discharge, and the storage within the saturated zone. The application of UZF in our models only was used to simulate the trade off between infiltration and groundwater discharge, based on the elevation of the water table relative to the topography.

Below sea level, two General Head Boundaries (GHBs) were imposed on the model for both free surface and prescribed head formulations. First, along the inundated portion of the topography (i.e., bathymetry), each model cell in the top layer was assigned a head equal to sea level at a given time step with a prescribed seawater concentration of $C_s$ = 35 g/L. Along the seaward, left vertical boundary, the second GHB was assigned to the first column, all layers of the model with a prescribed $C_s$ = 35 g/L, and the head equal to the sea level elevation. The conductance for all GHB cells was calculated using the cell dimensions and its isotropic hydraulic conductivity following the standard definition (e.g., Eq 2-4) in^62^.

The transport of salt solutes was simulated below the water table using the Transport component of MODFLOW 6^68^. To consider the effect of salinity on flow, the seawater salinity was converted to density using a starting density of 1000 kg/m$^3$ for 0 g/L and 0.7 kg/m$^3$ for each additional g/L (i.e., rate of increase in density as a function of concentration), thus accounting for density dependence. The flow and transport models were solved separately for each time step (i.e., not using a single couple matrix), such that each next flow time step used the concentrations solved in the previous time step^68^. Advective transport was solved using the upstream weighting scheme, which translates cell concentration to the cell faces based on adjacent concentrations considering flow direction. To constrain solute dispersion, we assigned the effective molecular diffusion coefficient as 0 ($m^2/day$), the longitudinal dispersivity in the horizontal and vertical directions as 1 m, and the transverse dispersivity in both direction as 0.1 m. No decay or sorption were included in the representation of the salinity dynamics, thus only dilution of seawater by fresh groundwater recharge due to mixing was included in this modeling framework. For the spin-up models, the initial concentration was set to 0 mg/L for each scenario. The last time step of the spin-up model was used to set the initial concentration distribution in the main model run. In the context of solute transport, the intricate interplay of dispersion and diffusion processes introduces complexities that limited the application of nondimensionalization^69–71^.

The simulations were performed in two time-dependent stages. First, we ran a 30-year spin-up model to stabilize the saline wedge without sea-level rise. Second, we ran the models for 100-year periods with a constant rate of sea-level rise. Both model stages used a primary time step or stress period of one month, not accounting for seasonal nor tidal transience. The last time step from the spin-up model supplied hydraulic head and concentration distributions as the initial conditions for the sea-level rise simulations. An annual sea-level rise rate of 3.6 mm was used for all models, representing the global mean^4^. The performance of barriers for each sea-level rise model was evaluated at the last time step after 100 years of sea-level rise, amounting to 0.36 m. Coastal areas experiencing more or less extreme rates of sea-level rise can still use the results of this study as long as the groundwater system responds quickly enough to the sea-level change, although the timing of barrier performance would have to shift accordingly.

### Subsurface barrier implementation

We simulated numerous groundwater flow scenarios to understand the influence of subsurface barriers on the flow system and seawater intrusion. First, we conducted simulations without barriers to provide a basis for quantifying how introducing barriers alters the saline groundwater wedge and flow system. Next, we modeled the domain with a subsurface dam or a cutoff wall with barriers set as impermeable, no-flow cells (Fig. 1a, b).

Two parameters describing the barrier construction were tested in the study. First, the relative opening ($D^*$) of the aquifer at the barrier describes the amount of the aquifer remaining unimpeded:

1 $$\begin{aligned} D^* = \frac{D_{opening}}{D_{aquifer}}, \end{aligned}$$

with $D_{aquifer}$ the thickness of the aquifer at the barrier, $D_{opening}$ the opening length above or below the barrier, and $D_{barrier}$ the height of the barrier (Fig. 1c,d). While $D_{ocean}$ is set to a constant thickness of 50 m in the models (Fig. 1c, d), $D_{aquifer}$ varies according to the *S* in the model. $D^*$ therefore normalized the size of the aquifer opening between models with different slopes to allow direct comparison. Model values for $D^*$ were set to 0.1–0.9 with an interval of 0.1. Small values of $D^*$ indicate more extensive barrier construction and thus a shorter aquifer opening. Second, the relative location, ($L^*$), of the barrier from the shoreline is:

2 $$\begin{aligned} L^* = \frac{L_{barrier}}{L_{domain}}, \end{aligned}$$

with $L_{barrier}$ the horizontal location of the barrier relative to the shoreline at the starting sea level and $L_{domain}$ the total domain length (1000 m). Thus, for scenarios with $L^*=0$ or low slopes, the barrier becomes inundated by sea-level rise by the end of the simulation. Model values of $L^*$ were set to 0, 0.03, 0.05, 0.1, 0.15, 0.2, and 0.3, where the focus of constructing protective barriers is expected to occur relatively close to the present-day shoreline.

### Evaluation indicators

Previous studies addressing groundwater responses to subsurface barriers mainly focus on the salt wedge reduction ratio^27,45^, which we relabel as the seawater intrusion reduction ratio, $R_{intrusion}$ (defined mathematically below), to clarify the groundwater focus of this barrier performance indicator. Additional work has quantified how a subsurface barrier affects the subsurface flux partitioning or may influence submarine groundwater discharge^43^. Moreover, other intrusion indicators from other studies include the amount of salt mass reduction provided by the construction of a subsurface barrier^27^ and the reduction in width of the saline-fresh mixing zone^45^. To our knowledge, no work has quantified how terrestrial groundwater discharge or groundwater emergence are influenced by a subsurface barrier. For a comprehensive evaluation of groundwater-oriented performance of the subsurface barriers, three indicators were used in our analysis: (1) the seawater intrusion reduction ratio, $R_{intrusion}$, (2) the inland flow ratio, $R_{flow}$, and (3) the inland groundwater emergence ratio, $R_{emergence}$. Thus, we introduce two additional performance indicators, $R_{flow}$ and $R_{emergence}$, focused on quantifying the flow and water table level conditions altered by subsurface barriers that have been acknowledged but overlooked as important hydrologic features in previous work.

In this study, $R_{intrusion}$ is defined as:

3 $$\begin{aligned} R_{intrusion}= \frac{L_{SW,0} - L_{SW,i}}{L_{SW,0}} \end{aligned}$$

with $L_{SW,i}$ the final simulated horizontal length of the salt wedge toe from the starting horizontal location of sea level for a scenario, *i*, with a barrier. The location of the saline-fresh interface was defined using the 1.4% salt concentration contour, a drinking water threshold for freshwater^72^. $L_{SW,0}$ was the calculated salt wedge length without a barrier with all other parameters identical. $R_{intrusion}=1$ identifies a fully protective barrier with no salt wedge inland of the barrier, while $R_{intrusion}=0$ indicates no protective benefit of the barrier.

To quantify how the subsurface barrier partitions groundwater flow, the inland groundwater flow ratio, $R_{flow}$, was defined as:

4 $$\begin{aligned} R_{flow} = \frac{Q_{inland}}{Q_{inland} + Q_{ocean}} = \frac{Q_{inland}}{r}, \end{aligned}$$

with $Q_{inland}$ the inland or upstream fresh groundwater discharge occurring at the land surface upland of the barrier and $Q_{ocean}$ the groundwater flow through the opening above or below the barrier (Fig. 1a, b). $R_{flow}=0$ indicates all groundwater discharges seaward of the barrier, while increasing values indicate discharge inland of the barrier with $R_{flow}=1$ indicating all groundwater inland of the barrier discharges at or upland of the barrier. In our model formulation, $Q_{inland}$ can only flow from areas with groundwater emergence.

High $R_{flow}$ indicates relatively more fresh groundwater discharge inland of the barrier in areas with groundwater emergence, but the implications of this discharge on the extent of groundwater emergence requires an additional metric. To quantify how subsurface barriers affect groundwater emergence, we introduce $R_{emergence}$ as:

5 $$\begin{aligned} R_{emergence} = \frac{L_{emergent}}{L_{inland}}, \end{aligned}$$

with $L_{emergent}$ the length of the land surface with emergent groundwater and $L_{inland}$ the total length of the domain inland of the barrier. Cells were considered emergent and their widths added to $L_{emergent}$ if the modeled head was $\le$ 5 cm below the land surface elevation to account for the use of subgrid surface depressions in the UZF package. $R_{emergence}$ was dependent on the lateral discretization of the model, where one cell width was the minimum $L_{emergent}$ numerator (i.e., 5 m for this study).

We expect that an effective or successful subsurface barrier should result in a groundwater flow condition that results in a high $R_{intrusion}$ (i.e., prevent more salt water intrusion), low or zero $R_{flow}$ (i.e., causing less inland fresh groundwater discharge), and low to zero $R_{emergence}$ (i.e., causing less inland groundwater emergence). However, the ultimate definition of a successful or effective barrier depends on site-specific stakeholder needs and agreed upon trade-offs between these ratios, discussed further in the following sections.

### Analysis limitations

We adopted a simple, two-dimensional cross-section framework to investigate the fundamental controls of subsurface barriers on groundwater partitioning, groundwater emergence, and saltwater intrusion with sea-level rise, but additional complexities could be tested. For example, our models used a constant topographic slope with a flat aquifer bottom to test how changes in transmissivity caused by aquifer thickness, changing geology via *K*/*r*, and barrier construction changed partitioning and intrusion. More complex geologic heterogeneity could be investigated with sloping geology^19^, additional geologic contacts and/or geostatistical representations^20,73^, or by using more variable topography. Additionally, subsurface barriers could be simulated in three-dimensions, where the alongshore extent of hydrologic changes affected by the barriers could be characterized^74^. We also only simulated single barriers as completely impermeable, where both combinations of barrier types^28,46^ and semi-impermeable barriers have been considered in other studies^27^ without the focus on groundwater partitioning and emergence. Furthermore, the barriers in the model were a uniform width, one column in the model or 5 m wide, and the width of the barrier could influence the flow partitioning as it sets the length over which the head drop induced by the barrier occurs. Our models also did not include other human impacts on aquifer processes, where interactions with pumping^13^ or sewer infrastructure^75^ could create additional hydrogeologic complexity for quantifying shallow aquifer responses to the construction of subsurface barriers and alter intrusion and emergence behavior. Finally, our model uses a robust but simple representation of groundwater-surface water interactions through the GHB ocean boundary and with the internal water balance of recharge and discharge across the land surface with UZF. A more complex formulation could include groundwater-surface water interactions that allow groundwater discharge to flow overland, pond up and create a backwater effect, and/or once again recharge into the subsurface. While not considering many of the complexities that could be expected for a specific coastal site, our study highlights the importance of how subsurface barriers alter groundwater emergence and groundwater flow, could affect their performance to protect against seawater intrusion, and could create inland groundwater flooding hazards.

Importantly, our simulation framework tested only the protective trade-offs for simple unconfined aquifers. For topography-limited coastal aquifers, lateral groundwater flowpaths and the connection of the aquifer to the surficial drainage network would add complexity to barrier effectiveness^23^. Since a majority of coastal aquifers globally are topography-limited^22^, the degree to which a particular setting has shore-perpendicular groundwater flow should be carefully considered when applying the results of this study^74^. Further, these simulations only included relatively long-term but still fairly minor sea-level rise as the driver of seawater intrusion and groundwater flow changes. Shorter-term hydrodynamics and salinization of the aquifer could occur with the compounding effects of tides and storm surges^76^ or longer-term and higher rates of sea-level rise. These simulations rely on relatively simple hydrogeologic conditions and two-dimensional flow, where field observations and more complex models could identify additional challenges and solutions.

### Supplementary Information

Supplementary Information.

## Data Availability

The datasets used and/or analysed during the current study available from the corresponding author in the HydroShare, Supporting Data repository^77^.
