# Supplementary material for: Shoreline barriers may amplify coastal groundwater hazards with sea-level rise
Source: Sci Rep. 2024 Jul 6;14:15559. doi: 10.1038/s41598-024-66273-w (PMC11226656; doi:10.1038/s41598-024-66273-w)
Supplement: Supplementary file 1 — Supplementary Information. [file 41598_2024_66273_MOESM1_ESM.pdf]

Supporting Information for: "Shoreline barriers may amplify coastal groundwater hazards with sea-level rise"

Xin Su[1,2,*], **Kevin M. Befus**[1], **and Michelle A. Hummel**[3]

[1]University of Arkansas, Department of Geosciences, Fayetteville, AR, 72701, USA
[2]Arizona State University, School of Sustainable Engineering and the Built Environment, Tempe, AZ, 85281, USA
[3]University of Texas at Arlington, Department of Civil Engineering, Arlington, TX, 76019, USA
[*]xin.su.5@asu.edu

ABSTRACT

Subsurface barriers have been proposed to protect coastal aquifers from sea-level rise induced saline groundwater intrusion, but the potential for groundwater emergence near subsurface barriers remains unknown. Here, we investigated how changing groundwater flow conditions and emergence influence the protective performance of subsurface barriers with sea-level rise. We tested these subterranean consequences of sea-level rise for cutoff walls and subsurface dams with cross-shore groundwater flow and salt transport models, investigating how barrier design, aquifer properties, and hydrology control the potential for intrusion, emergence, and groundwater partitioning at the barrier with sea-level rise. We find that most subsurface infrastructure cannot prevent intrusion and emergence simultaneously. Subsurface dams spanning more than half of the aquifer thickness created emergence hazards for all scenarios tested. Cutoff walls were less effective at reducing saline intrusion for all opening sizes but could reduce the emergence potential compared to similar sized subsurface dams. Our results demonstrate the challenging trade-offs in mitigating the coastal groundwater hazards of saline groundwater intrusion and emergence with sea level rise, where pumping may be needed to minimize the potential for groundwater flooding inland of protective infrastructure that could intercept groundwater flow.

Simulated salt wedge distributions

Examples of the simulated salt wedge distribution are presented for cutoff walls (Figures 1- 4) and subsurface dams (Figures 5-8), using the UZF-based simulations. The salt wedges were substantially different across D^* (0.2, 0.5, 0.8) and L^* (0, 0.05, 0.1) scenarios at different aquifer dimensions, while changes in the salt wedge distributions for varying barrier opening size, salt wedge differences were limited. Two salt wedge delineations were set using different salinity thresholds based on salt concentrations, C. The first was $C = 17.5g/L$, representing 50% of the salinity of seawater, and the second was set as $C = 0.45g/L$, representing the drinking water standard. These cross sections show the water table position and groundwater salinity distribution at the last time step for the model.

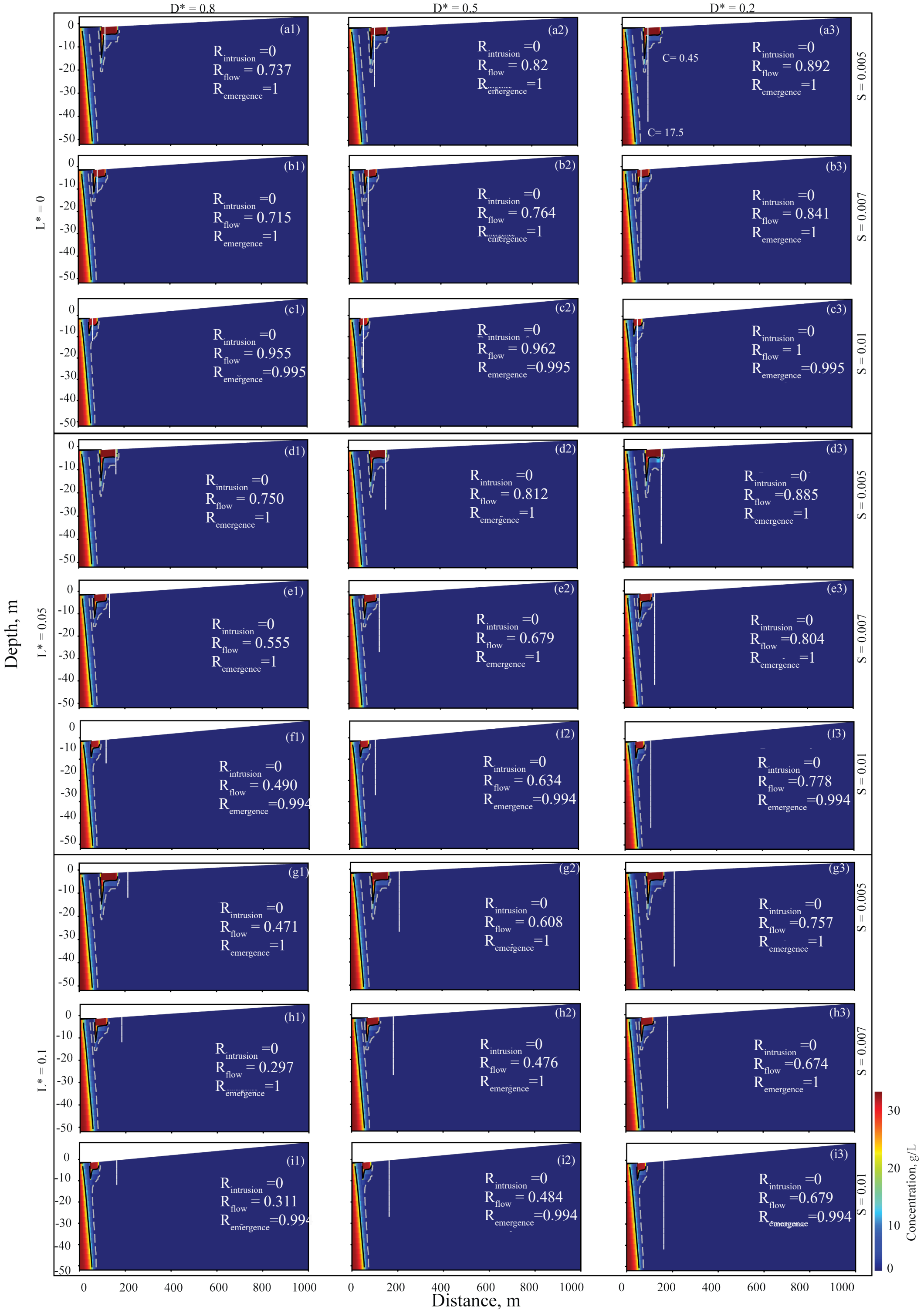

Figure S 1. Modeled salt wedge distributions for a cutoff wall with $K/r = 18$. Columns are labeled by the D^* (column 1 is $D^* = 0.8$, column 2 is $D^* = 0.5$, and column 3 is $D^* = 0.2$) used for the modeling, and each section (include 3 rows) is labeled by the barrier relative location, L^* (part a, b, c are $L^* = 0$, part d, e, f are $L^* = 0.05$, and part g, h, i are $L^* = 0.1$). Each row in one section labeled by the topographic slope (S). Each simulated case includes the calculated $R_{intrusion}$, R_{flow}, and $R_{emergence}$.

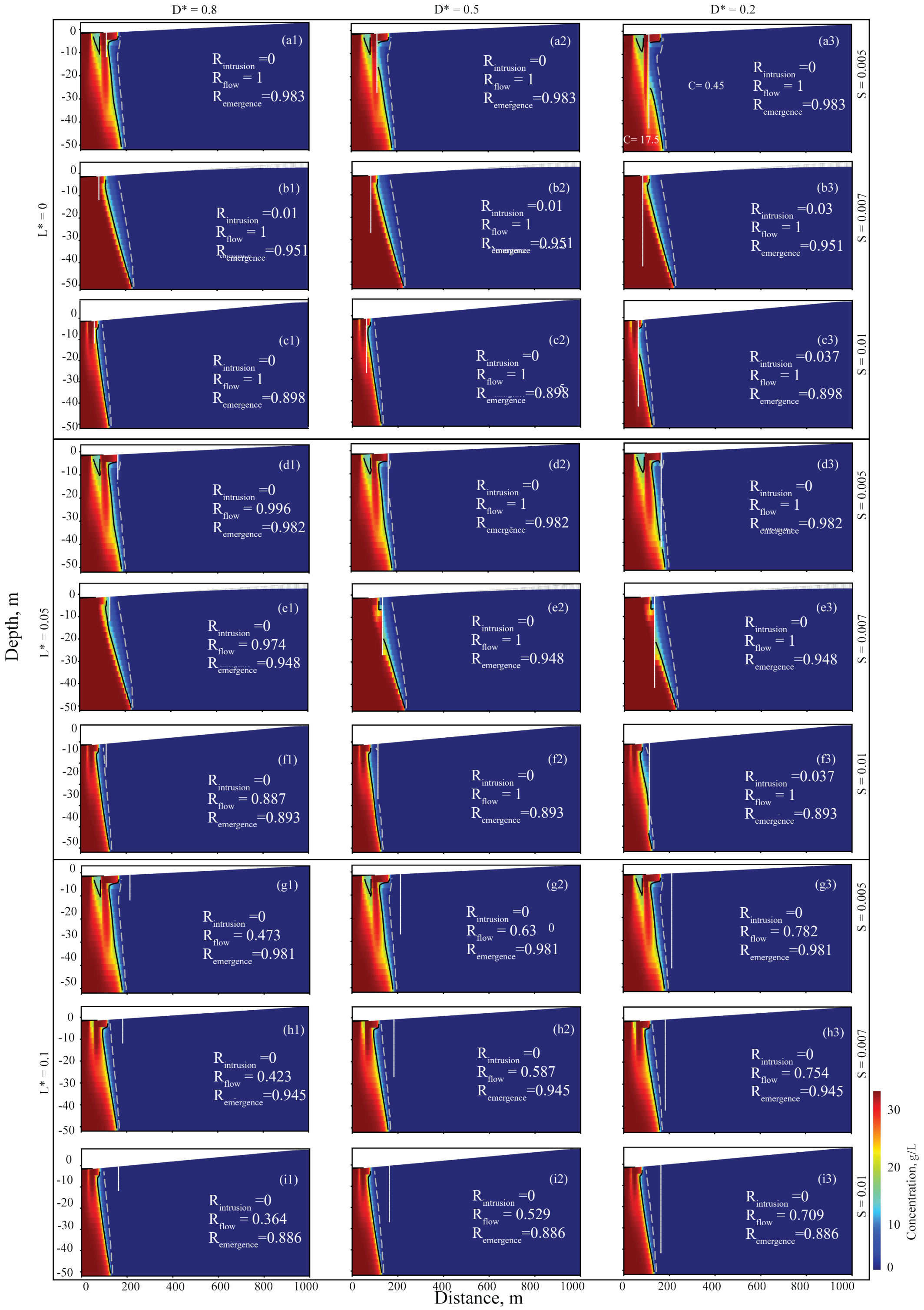

Figure S 2. Modeled salt wedge distributions simulated for a cutoff wall with $K/r = 180$. Columns are labeled by the D^* (column 1 is $D^* = 0.8$, column 2 is $D^* = 0.5$, and column 3 is $D^* = 0.2$) used for the modeling, and each section (include 3 rows) is labeled by the barrier relative location, L^* (part a, b, c are $L^* = 0$, part d, e, f are $L^* = 0.05$, and part g, h, i are $L^* = 0.1$). Each row in one section represents the topographic slope. Each simulated case includes the calculated $R_{intrusion}$, R_{flow}, and $R_{emergence}$.

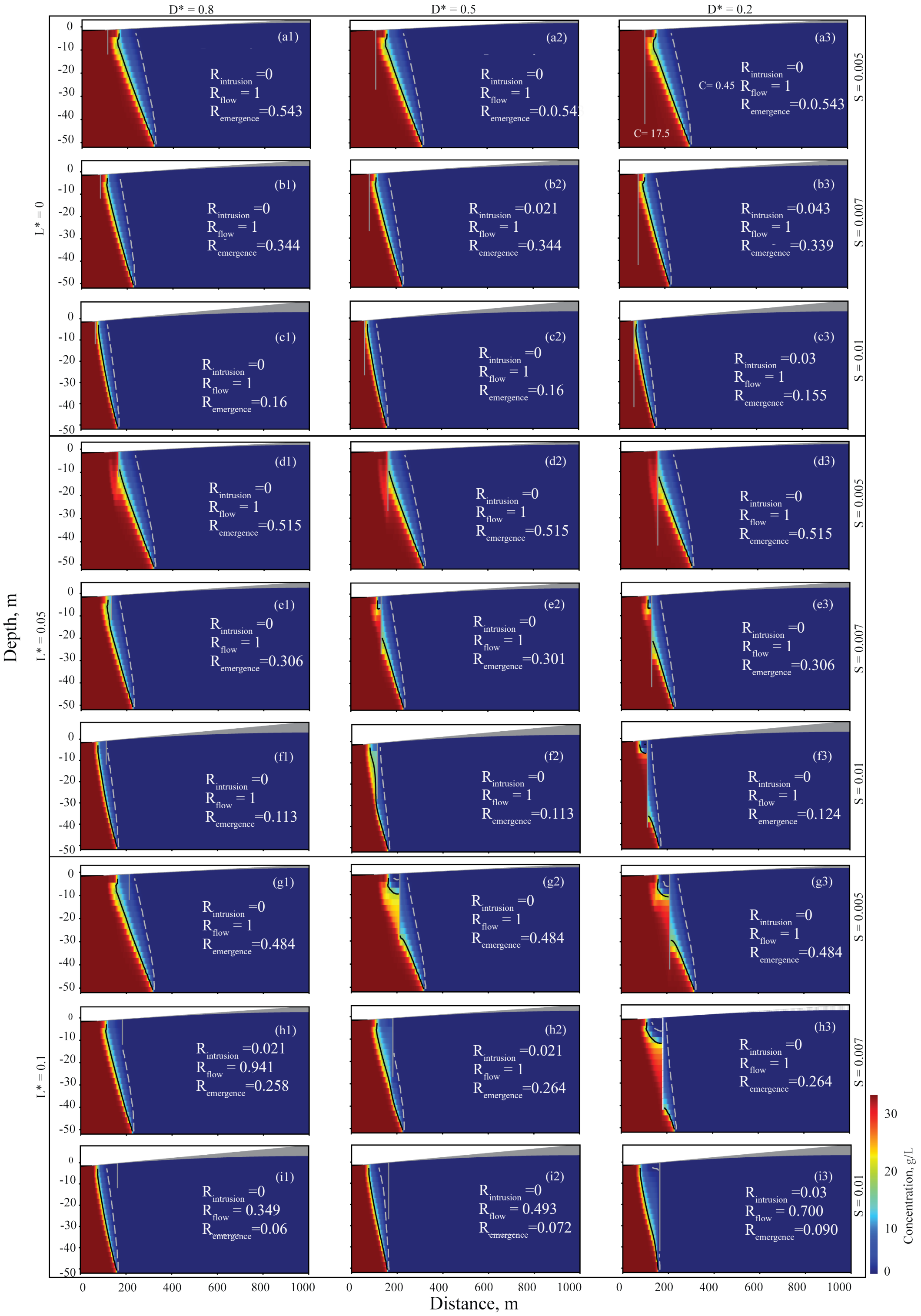

Figure S 3. Modeled salt wedge distributions simulated for a cutoff wall with $K/r = 1,800$. Columns are labeled by the D^* (column 1 is $D^* = 0.8$, column 2 is $D^* = 0.5$, and column 3 is $D^* = 0.2$) used for the modeling, and each section (include 3 rows) is labeled by the barrier relative location, L^* (part a, b, c are $L^* = 0$, part d, e, f are $L^* = 0.05$, and part g, h, i are $L^* = 0.1$). Each row in one section represents the topographic slope. Each simulated case includes the calculated $R_{intrusion}$, R_{flow}, and $R_{emergence}$.

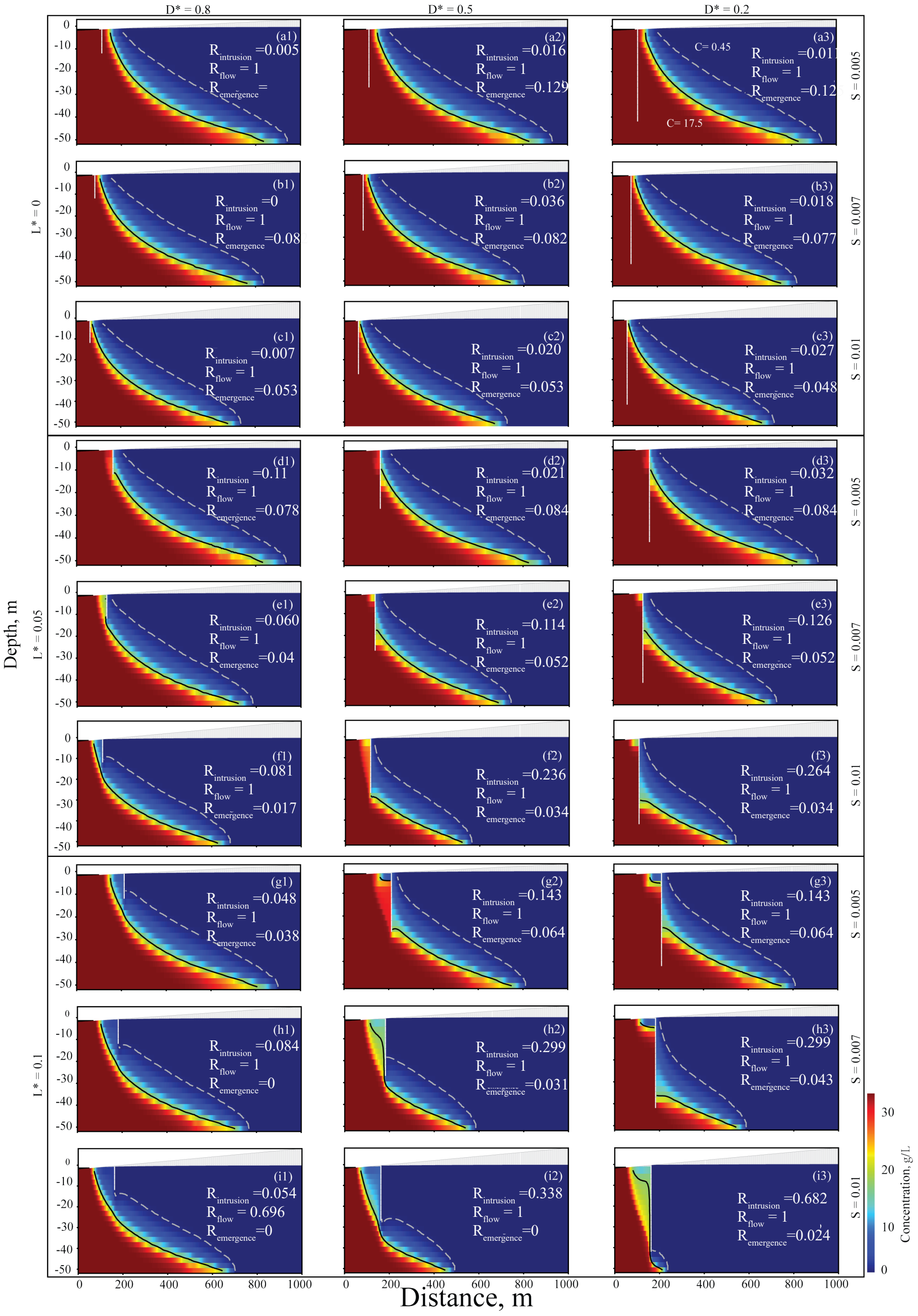

Figure S 4. Modeled salt wedge distributions simulated for a cutoff wall with $K/r = 18,000$. Columns are labeled by the D^* (column 1 is $D^* = 0.8$, column 2 is $D^* = 0.5$, and column 3 is $D^* = 0.2$) used for the modeling, and each section (include 3 rows) is labeled by the barrier relative location, L^* (part a, b, c are $L^* = 0$, part d, e, f are $L^* = 0.05$, and part g, h, i are $L^* = 0.1$). Each row in one section represents the topographic slope. Each simulated case includes the calculated $R_{intrusion}$, R_{flow}, and $R_{emergence}$.

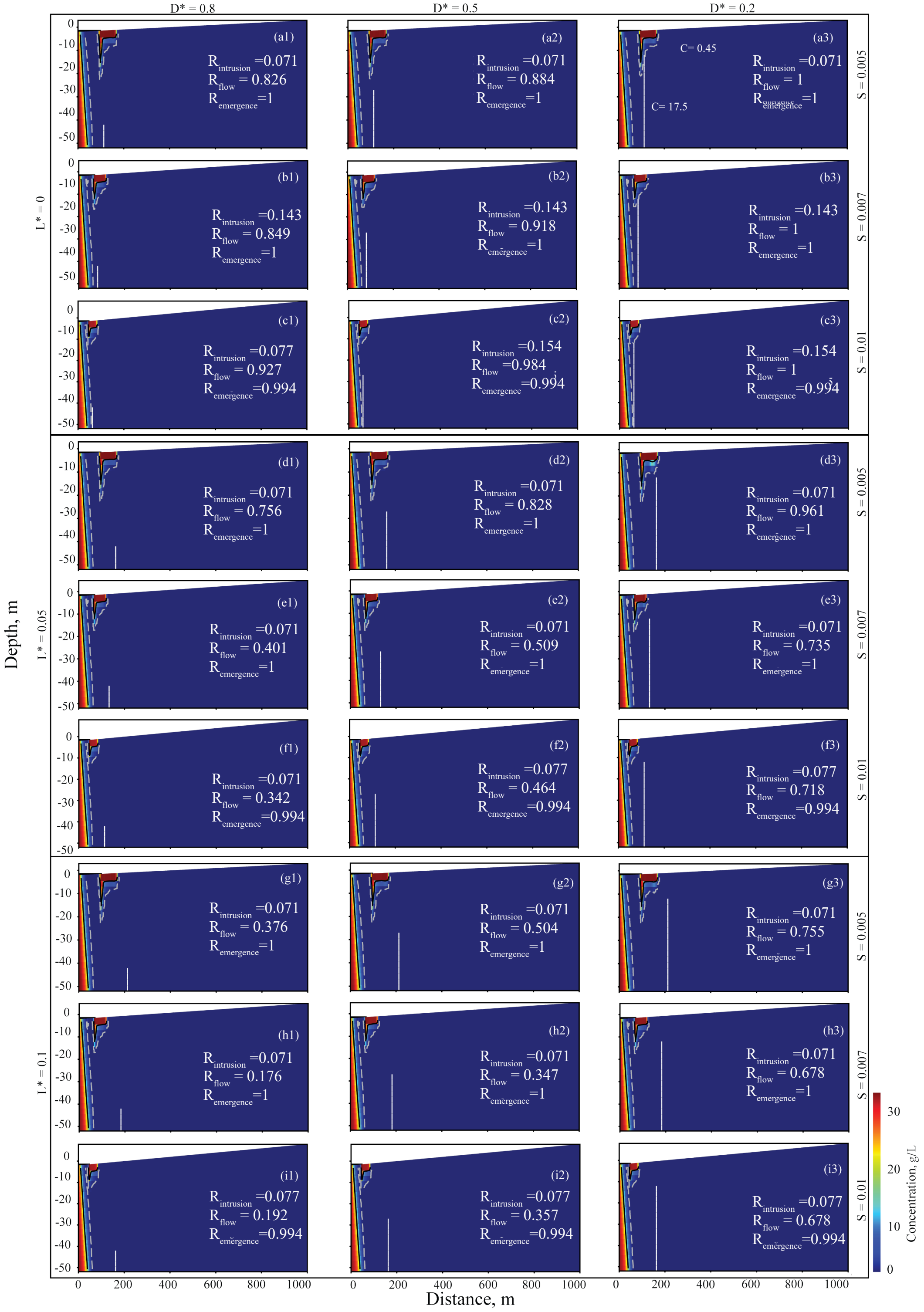

Figure S 5. Modeled salt wedge distributions simulated for a subsurface dam with $K/r = 18$. Columns are labeled by the D^* (column 1 is $D^* = 0.8$, column 2 is $D^* = 0.5$, and column 3 is $D^* = 0.2$) used for the modeling, and each section (include 3 rows) is labeled by the barrier relative location, L^* (part a, b, c are $L^* = 0$, part d, e, f are $L^* = 0.05$, and part g, h, i are $L^* = 0.1$). Each row in one section represents the topographic slope. Each simulated case includes the calculated $R_{intrusion}$, R_{flow}, and $R_{emergence}$.

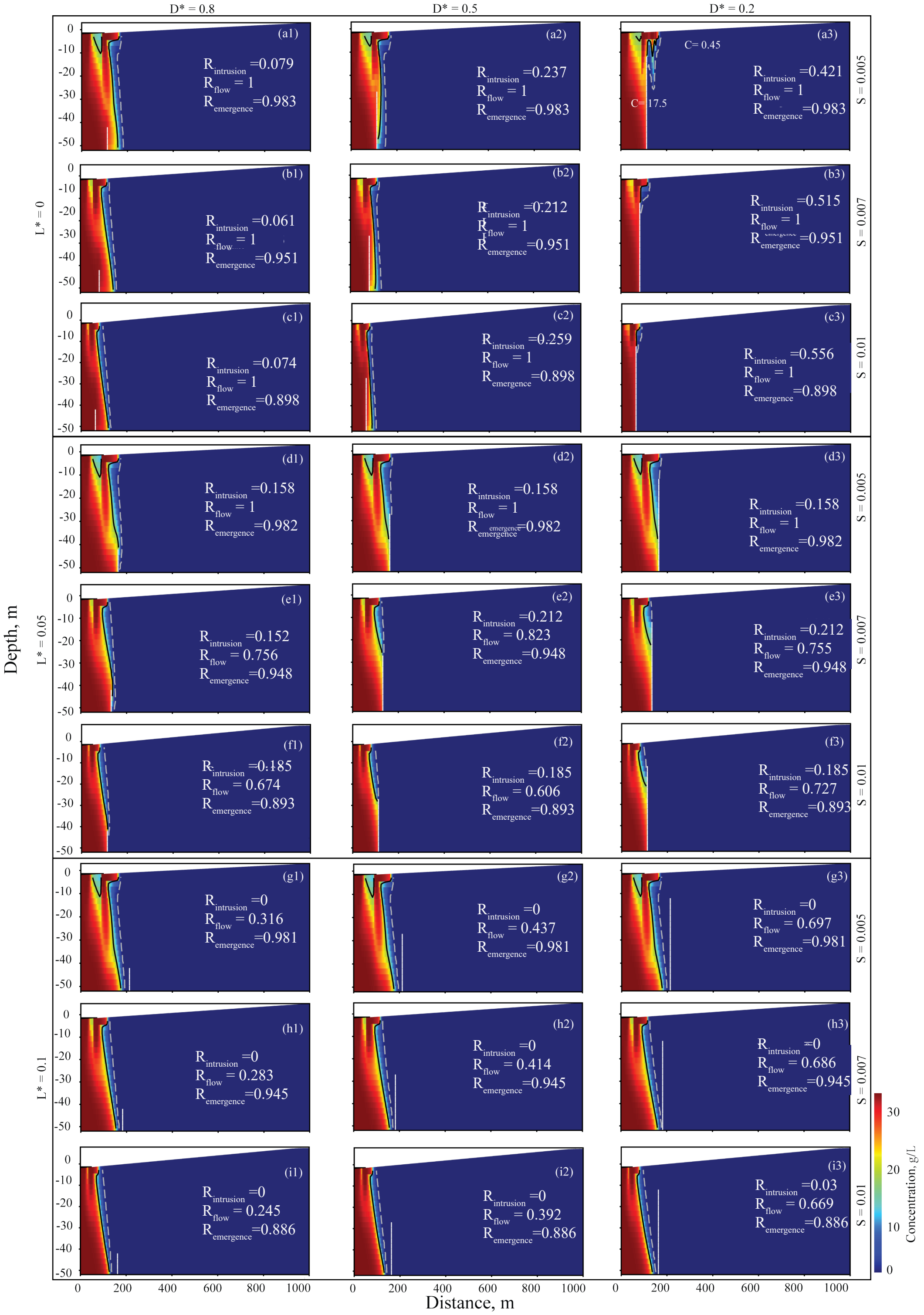

Figure S 6. Modeled salt wedge distributions simulated for a subsurface dam with $K/r = 180$. Columns are labeled by the D^* (column 1 is $D^* = 0.8$, column 2 is $D^* = 0.5$, and column 3 is $D^* = 0.2$) used for the modeling, and each section (include 3 rows) is labeled by the barrier relative location, L^* (part a, b, c are $L^* = 0$, part d, e, f are $L^* = 0.05$, and part g, h, i are $L^* = 0.1$). Each row in one section represents the topographic slope. Each simulated case includes the calculated $R_{intrusion}$, R_{flow}, and $R_{emergence}$.

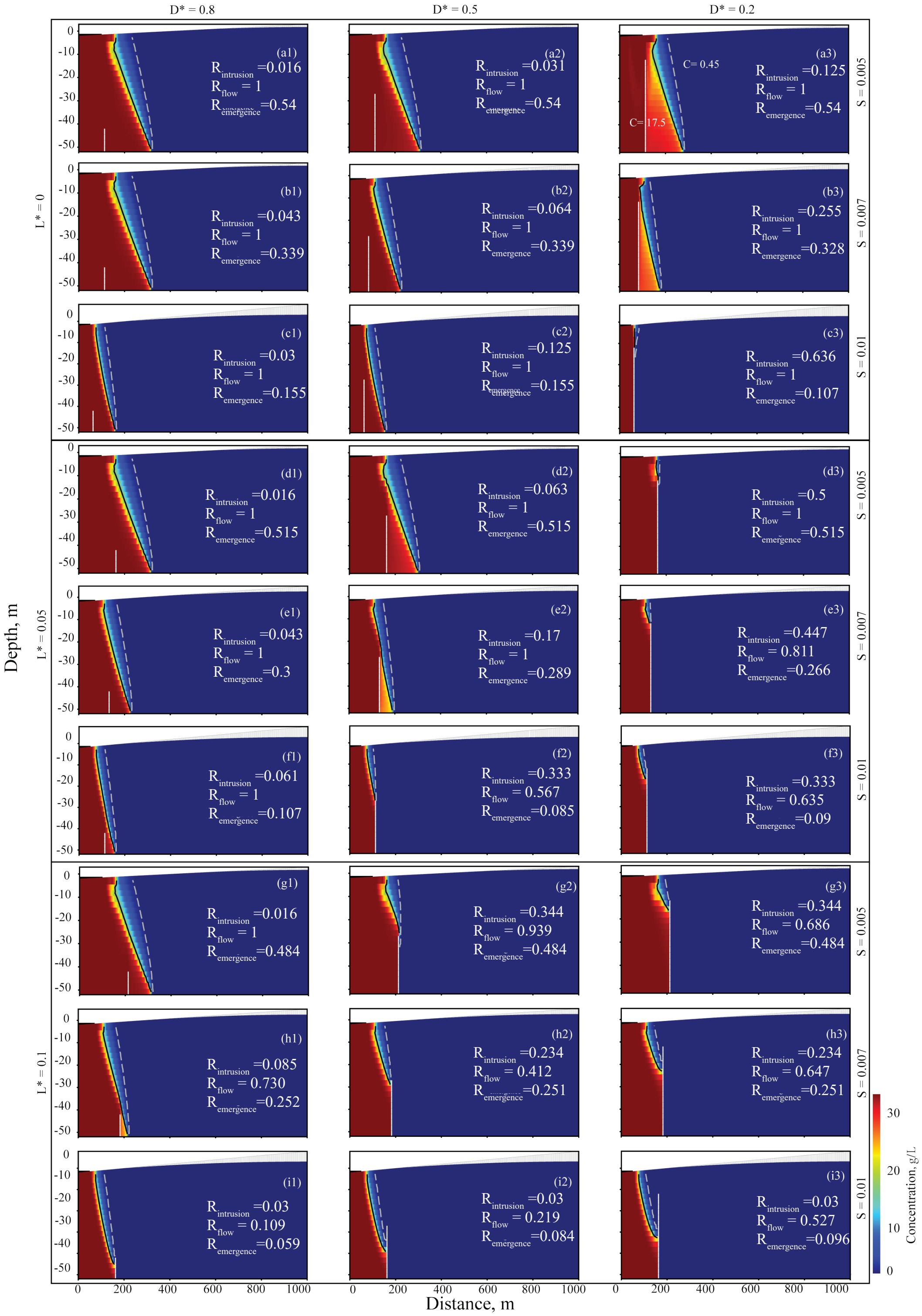

Figure S 7. Modeled salt wedge distributions simulated for a subsurface dam with $K/r = 1,800$. Columns are labeled by the D^* (column 1 is $D^* = 0.8$, column 2 is $D^* = 0.5$, and column 3 is $D^* = 0.2$) used for the modeling, and each section (include 3 rows) is labeled by the barrier relative location, L^* (part a, b, c are $L^* = 0$, part d, e, f are $L^* = 0.05$, and part g, h, i are $L^* = 0.1$). Each row in one section represents the topographic slope. Each simulated case includes the calculated $R_{intrusion}$, R_{flow}, and $R_{emergence}$.

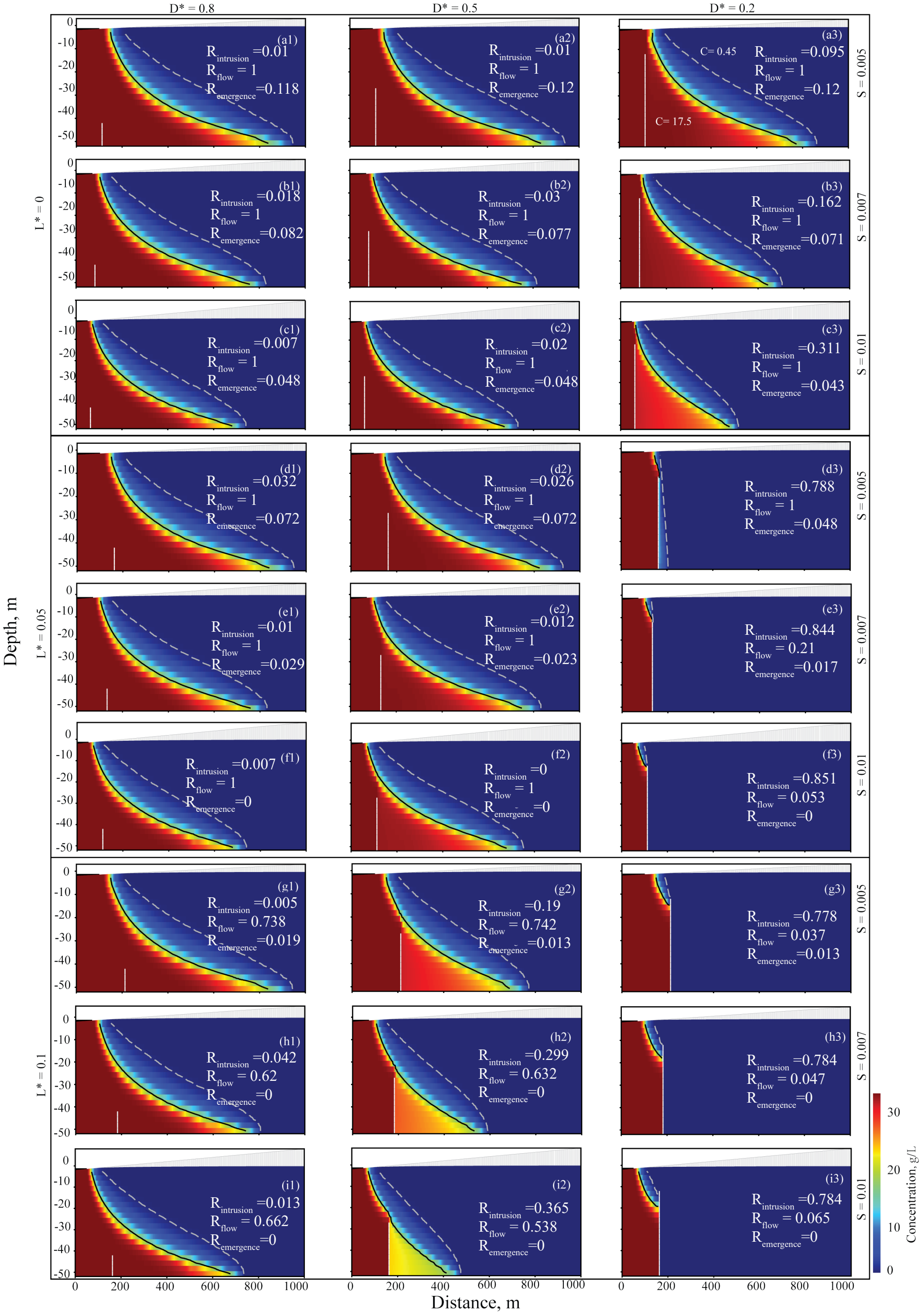

Figure S 8. Modeled salt wedge distributions simulated for a subsurface dam with $K/r = 18,000$. Columns are labeled by the D^* (column 1 is $D^* = 0.8$, column 2 is $D^* = 0.5$, and column 3 is $D^* = 0.2$) used for the modeling, and each section (include 3 rows) is labeled by the barrier relative location, L^* (part a, b, c are $L^* = 0$, part d, e, f are $L^* = 0.05$, and part g, h, i are $L^* = 0.1$). Each row in one section represents the topographic slope. Each simulated case includes the calculated $R_{intrusion}$, R_{flow}, and $R_{emergence}$.

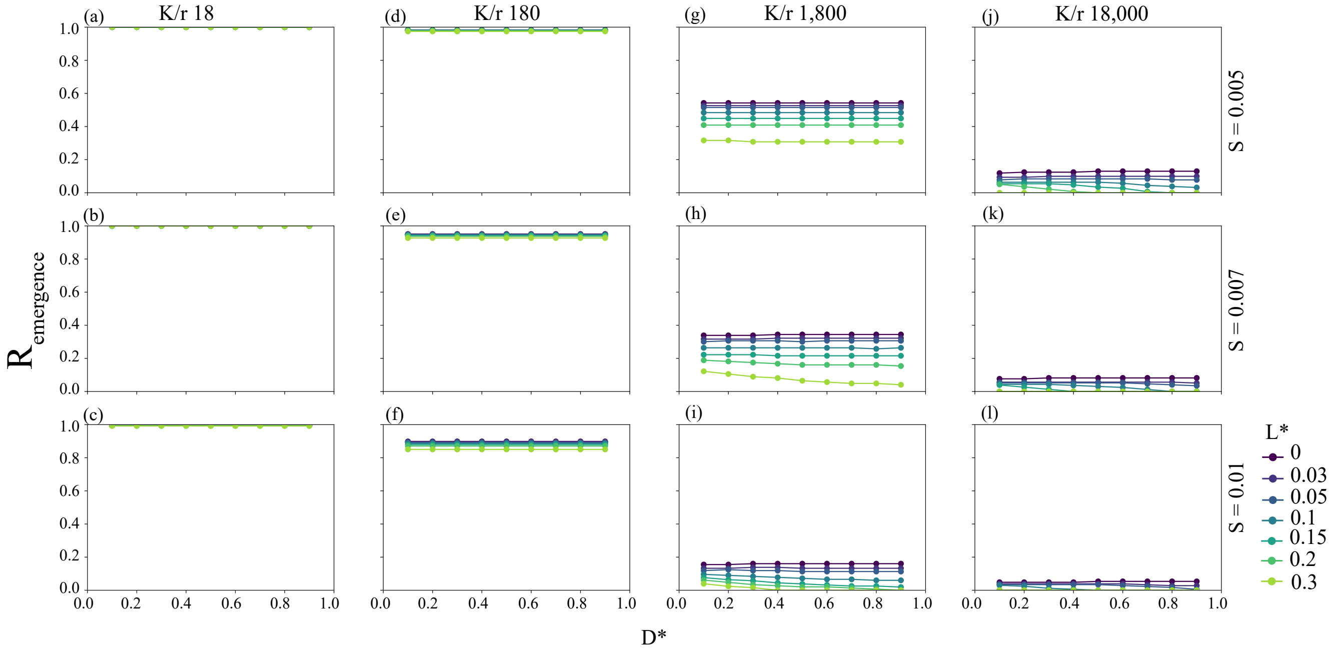

Figure S 9. $R_{emergence}$ simulated using the free surface formulation (i.e., UZF) for a cutoff wall. Columns are labeled by the K/r used for the modeling, and the rows are labeled by the topographic slope, S. Each point represents two simulations used to calculate $R_{emergence}$, one without a barrier and one with a barrier.

Evaluation indicator results

Barrier effects on inland emergence, $R_{emergence}$

The simulated $R_{emergence}$ values varied by up to 20% over barrier opening and barrier location scenarios for both cutoff walls and subsurface dams (Figures 9 and Figures 10). With $K/r \leq 180$, the system is heavily topography-controlled, which lead to almost the entire inland land surface with emergence ($R_{emergence} > 0.88$ for subsurface dams and $R_{emergence} > 0.85$ for cutoff walls). Changing the barrier depth and location did not alter the emergence conditions, indicating less overall barrier influence relative to the hydrogeologic parameters. However, with larger K/r, the barrier opening and location played a more important role, especially for $K/r = 1,800$ scenarios (in Figures 9 g-i and 10 g-i). In our simulations, by increasing the relative opening, most of the tested scenarios did not have significant responses in $R_{emergence}$. However, for the most inland barriers with $K/r = 1,800$, the $R_{emergence}$ decreased up to 0.2 from $D* = 0.1$ to $D* = 0.9$. While for $K/r = 18,000$ conditions, inland groundwater was more efficiently drained and discharged through the aquifer, which highly reduces the opportunities for significant discharge inland of the barrier, especially for low subsurface dams or cutoff walls that did not intersect the water table. At $K/r = 18,000$, the most severe emergence occurred for both a cutoff wall and subsurface barrier with $R_{emergence} = 0.13$. For all tested aquifer conditions for both barriers, the surface slope also controlled the overall occurrence of inland emergence, where the steeper the surface slope was the less inland emergence occurred. While many scenarios resulted in very little change in $R_{emergence}$ with changes in the barrier size (i.e., D^*), the interpretation that the barrier was having little influence on groundwater levels and flow required additional analysis of the $R_{intrusion}$ and R_{flow} to fully understand how the barrier affected the groundwater system.

Barrier effects on intrusion protection, $R_{intrusion}$

The simulated $R_{intrusion}$ varied most with K/r = 1,800 and 18,000 followed by D^* for cutoff walls and subsurface dams (Figure 13 and Figure 14). $R_{intrusion}$ was effectively zero for $K/r \leq 1,800$ for cutoff walls (Figure 13a-i). With $K/r \leq 1,800$ for subsurface dams, the $R_{intrusion}$ varied substantially (Figure 14a-i). At lower K/r (K/r = 18, 180), topography-controlled conditions resulted in low $R_{intrusion}$ with the wedge intruding inland of the barrier. For these low K/r conditions, a subsurface dam overall prevented more saltwater intrusion than a cutoff wall for similar parameter combinations. It is important to note that $K/r = 18$ for the subsurface dam led to instability in the saline groundwater wedge seaward of the barrier such that the minimum $R_{intrusion}$ for these simulations was not 0 for any parameter combination (Figure 14a-c), while models with higher K/r were more likely to simulate no intrusion benefit from the barrier (i.e., $R_{intrusion} = 0$). Both barriers demonstrated interesting trends for $R_{intrusion}$ when varying D^* and L^* most noticeably for $K/r = 18,000$ (Figure 13j-l and 14j-l. $R_{intrusion}$ increased with

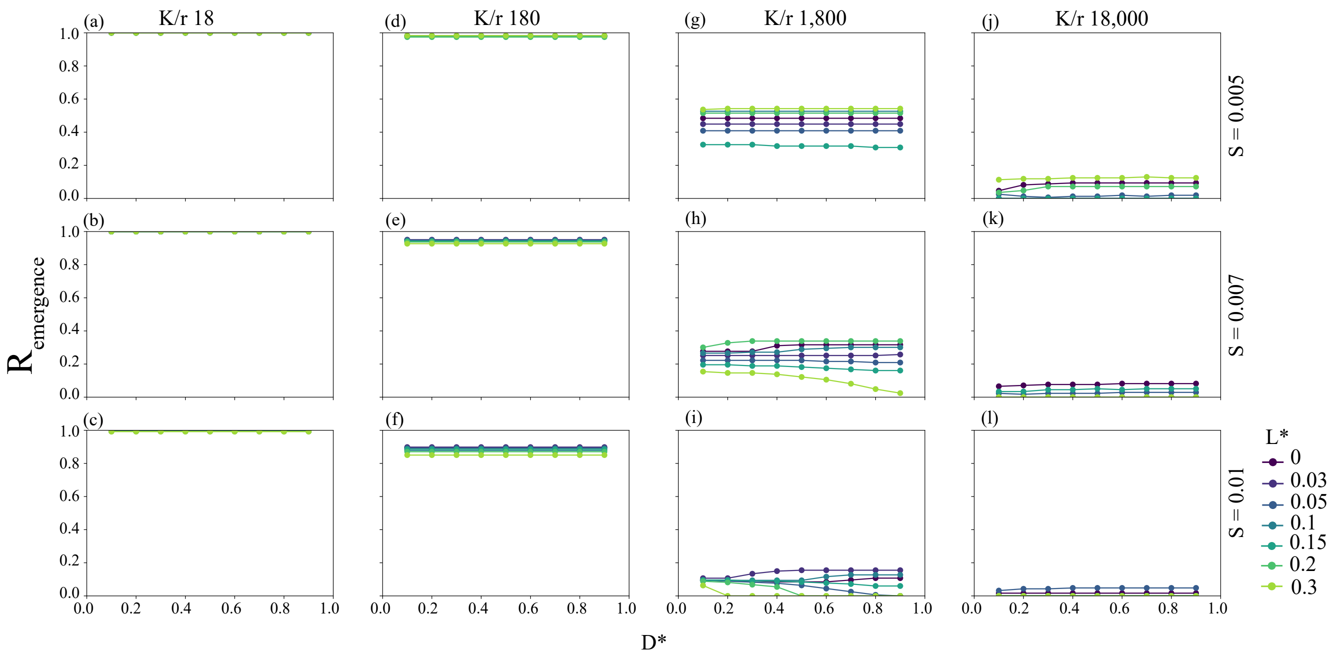

Figure S 10. $R_{emergence}$ simulated using the free surface formulation (i.e., UZF) for a subsurface dam. Columns are labeled by the K/r used for the modeling, and the rows are labeled by the topographic slope, S. Each point represents two simulations used to calculate $R_{emergence}$, one without a barrier and one with a barrier.

increasing L^* and constant D^*, reached a maximum around L^* = 0.15 for cutoff walls and 0.05 for subsurface dams, and then decreased with barriers farther inland, as high L^* barriers were farther from the coast and less likely to intersect the saline wedge. Increasing D^* shifted the $R_{intrusion}$ maximum to larger L^*. Maximum values of $R_{intrusion}$ were 0.71 for a cutoff wall (Figure 13l) for $D^* = 0.1$ and $L^* = 0.1$ and 0.91 for a subsurface dam (Figure 14l) for $D^* = 0.1$ and $L^* = 0$. Selected salt wedge distribution results are provided in the supporting information (Supplemental Figures S1 and S2).

Although the cutoff wall was relatively ineffective in blocking saline groundwater intrusion for low K/r conditions ($K/r \leq 1,800$), $R_{intrusion}$ became more influenced by D^* and L^* for $K/r = 18,000$ (Figure S13j, k, l). $R_{intrusion}$ decreased substantially with increasing D^* for these cutoff wall simulations, with the moderate $L^* = 0.1$ most sensitive to changing the aquifer opening. These conditions were caused by the cutoff wall forcing fresh groundwater to flow under the barrier and, as intended with building such infrastructure, deflect the saline-fresh interface seaward (Supplemental Figure S1-S4). Increasing D^* for cutoff wall simulations decreased the blockage of the underflow of fresh groundwater and limited the discharge inland of the barrier, overall reducing the intrusion protection and $R_{intrusion}$ (see Section). At low slopes ($S = 0.005$) and $K/r = 18,000$, several cutoff wall simulations with $L^* > 0$ resulted in the formation of two disconnected freshwater lenses (Supplementary Figure S1). The freshwater lenses seaward of the barriers were not considered in the calculation of $R_{intrusion}$ because only the intrusion inland of the barrier was used to calculate $L_{SW,i}$ and to maintain a consistent analysis domain.

Unlike the cutoff wall, the subsurface dam implementation resulted in higher $R_{intrusion}$ values for most scenarios, but similarly, $R_{intrusion}$ decreased with increasing D^* (e.g., Figure S14d-l). Similar to cutoff walls, $R_{intrusion}$ was effectively negligible for all parameter combinations with $K/r < 18,000$, where the subsurface dam was too far inland to influence saline intrusion. Comparing with the performance of cutoff walls in preventing saltwater intrusion, the maximum $R_{intrusion}$ reached 0.92 for $L^* = 0$ and $D^* = 0.1$ with the steeper topography (Figure S14l), which was larger than the 0.71 maximum for cutoff walls. The subsurface dam also prevented up to 56% of the saltwater intrusion at a lower K/r conditions ($K/r = 180$) (Figure S14f), while cutoff walls effectively offered no intrusion benefit. For lower $K/r = 18$ scenarios, the maximum $R_{intrusion}$ only reached 0.18 with steep topography and a large barrier ($D^* = 0.1$) (Figure S14c) but was effectively zero for cutoff walls. Finally, these results showed that $R_{intrusion}$ for subsurface dams was highly sensitive to the barrier height and placement, and saltwater intrusion occurred via overtopping when the subsurface dam was installed at $L^* = 0$ for lower slope conditions.

Cutoff wall models with $K/r < 1,800$ resulted in prescribed head (CHD) models predicting up to 20% less intrusion (i.e., larger $R_{intrusion}$) than the free surface (UZF) models (Figure 11g-i). With $K/r = 18,000$, CHD-based cutoff wall models predicted up to 60% lower $R_{intrusion}$ (maximum at $L^* > 0.15$)(Figure S13j-l), indicating a more inland pre-barrier saline wedge

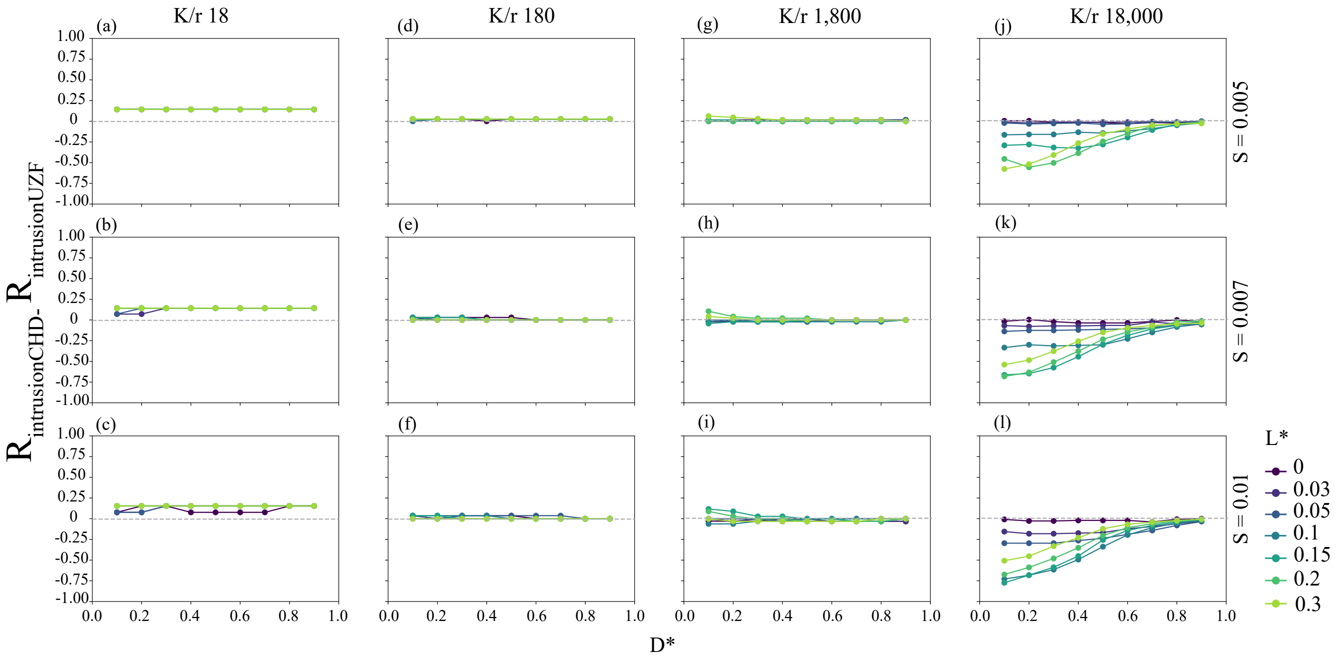

Figure S 11. Saline groundwater intrusion difference ratio ($R_{intrusion_{CHD}} - R_{intrusion_{UZF}}$) comparison between CHD and UZF top model boundary conditions with a cutoff wall implementation. Columns are labeled by the K/r used for the modeling, and the rows are labeled by the topographic slope, S. Each point represents four simulations, as two simulations are needed for calculating $R_{intrusion}$ for each CHD and UZF implementation.

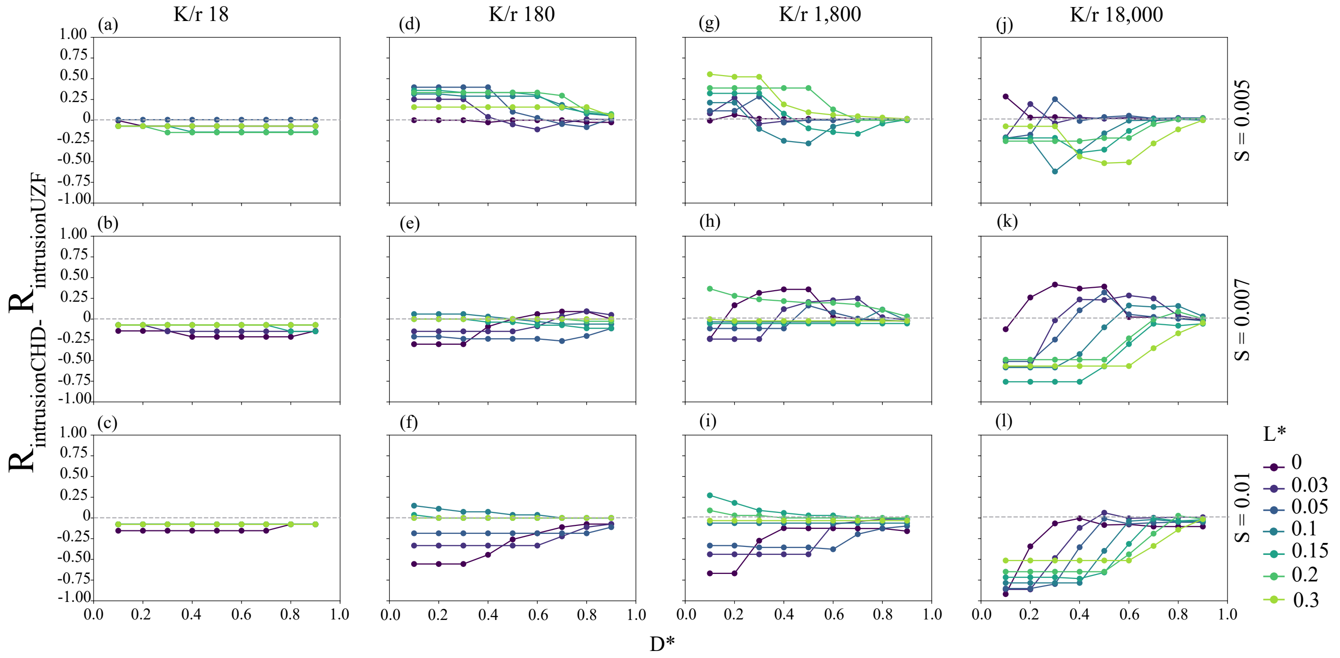

Figure S 12. Saline groundwater intrusion difference ratio ($R_{intrusion_{CHD}} - R_{intrusion_{UZF}}$) comparison between CHD and UZF top model boundary conditions with a subsurface dam implementation. Columns are labeled by the K/r used for the modeling, and the rows are labeled by the topographic slope, S. Each point represents four simulations, as two simulations are needed for calculating $R_{intrusion}$ for each CHD and UZF implementation.

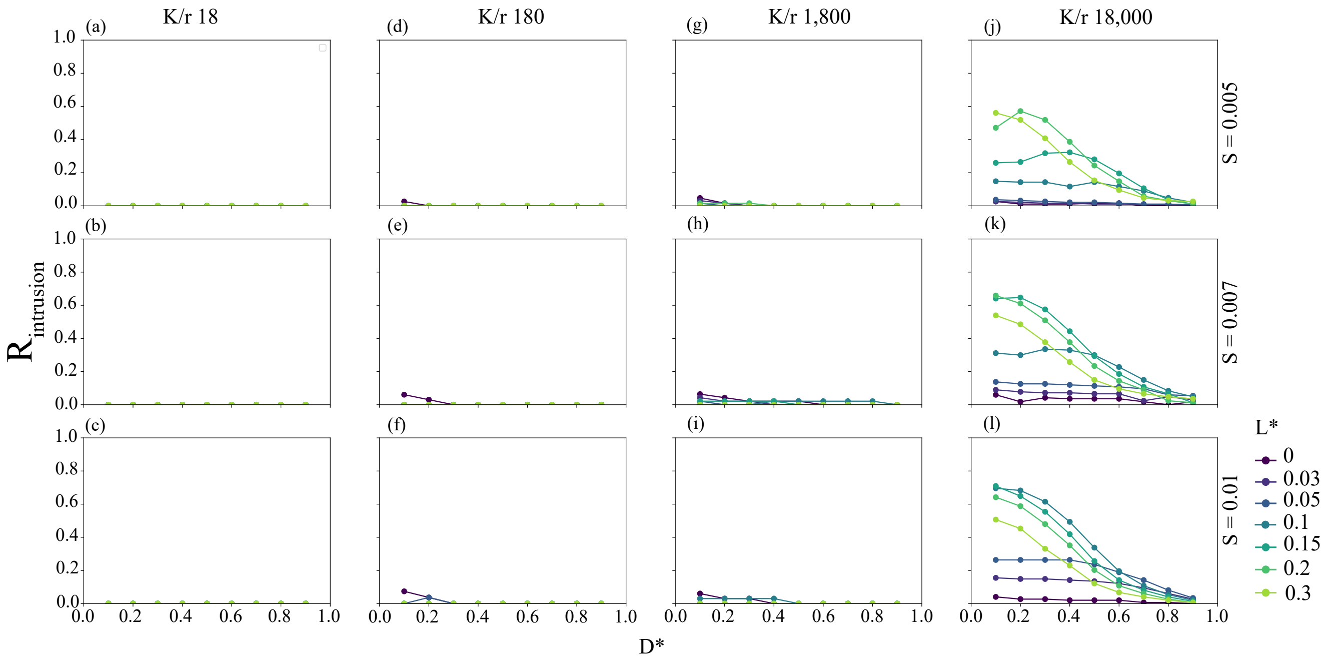

Figure S 13. Saline groundwater intrusion reduction ratio ($R_{intrusion}$) simulated for a cutoff wall. Columns are labeled by the K/r used for the modeling, and the rows are labeled by the topographic slope, S. Each point represents two simulations used to calculate $R_{intrusion}$, one without a barrier and one with a barrier.

by the UZF-based models. CHD-based $K/r \geq 180$ subsurface dam models also predicted lower $R_{intrusion}$ by no more than 50% because of a more extensive saline wedge in the UZF no-barrier models (Figure S12d-l).

Barrier effects on groundwater flow partitioning, R_{flow}

Overall, the R_{flow} under the cutoff wall scenarios increased with higher values of K/r simulated, especially for scenarios where $K/r < 18,000$ and $L^* < 0.15$. However, a threshold occurred between K/r values of 1,800 and 18,000, where the influence of the saltwater wedge position created an additional barrier contributing to larger R_{flow} at higher K/r. Thus, the groundwater flow partitioning for the cutoff wall with $K/r = 18,000$ was very sensitive to the evolving position of the fresh-saline interface, likely causing $R_{flow} = 1$ in the lower L^* scenarios. Additional increments of K/r would be required to further constrain this threshold across the parameter space, which was beyond the scope of the current analysis.

R_{flow} for the subsurface dam also decreased with larger values of L^*, indicating that more groundwater could flow over the subsurface dam as the barrier was built further inland. This effect was likely caused by the increasing aquifer thickness with distance inland set by the constant topographic slope (i.e., increasing transmissivity). Unlike the results from the cutoff wall cases, the R_{flow} results for the subsurface dam increased and then flattened with higher D^*, indicating the overtopping of the subsurface dam and onset of the saline groundwater wedge inland of the barrier controlling the flow partitioning. Under these flow conditions, a large portion of inland fresh groundwater discharge could initiate a hidden inland flooding risk by causing inland groundwater emergence[1].

Additional Supporting Information - Open Data

1. *readme.txt*: "Read me" file describing columns, units, and data provided in the following files.
2. *CutoffWall.csv*: R_{flow}, $R_{intrusion}$, and $R_{emergence}$ data for cutoff wall implementation at each aquifer dimensions and barrier conditions.
3. *Dam.csv*: R_{flow}, $R_{intrusion}$, and $R_{emergence}$ data for subsurface dam implementation at each aquifer dimensions and barrier conditions.
4. *Xsection.py*: example model script using FloPy to build and run MODFLOW 6 models.

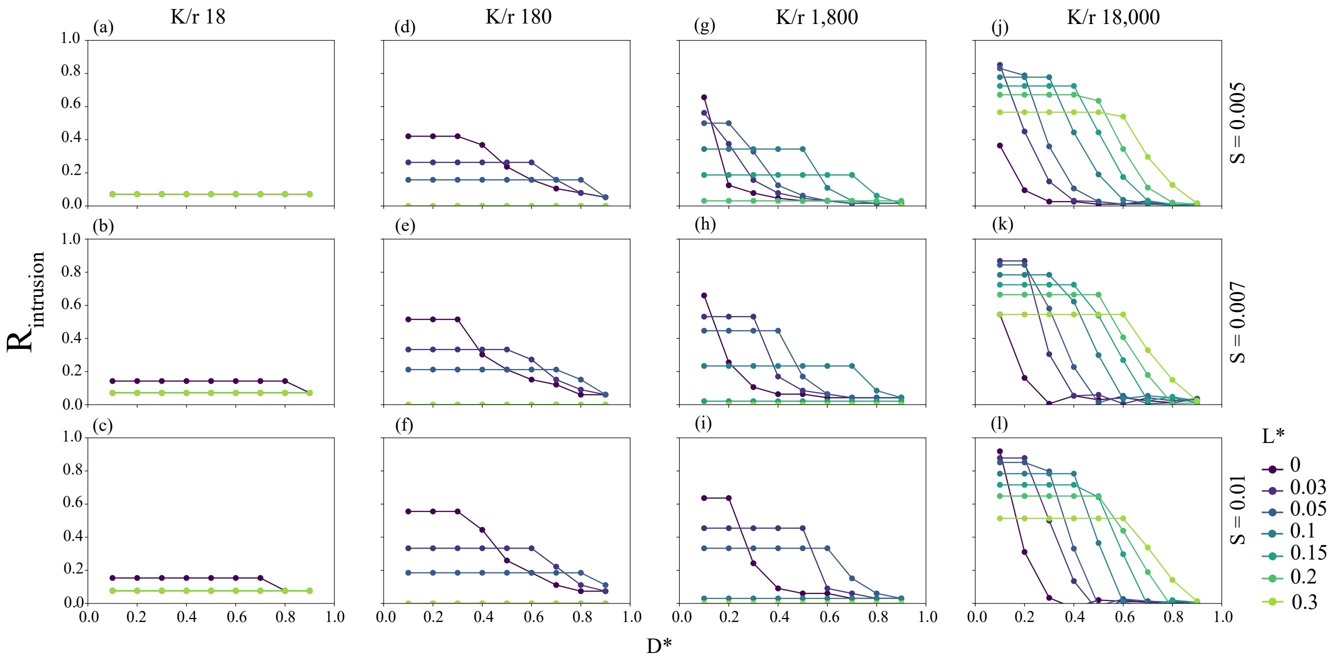

Figure S 14. Saline groundwater intrusion reduction ratio ($R_{intrusion}$) simulated for subsurface dam. Columns are labeled by the K/r used for the modeling, and the rows are labeled by the topographic slope, S. Each point represents two simulations used to calculate $R_{intrusion}$, one without a barrier and one with a barrier.

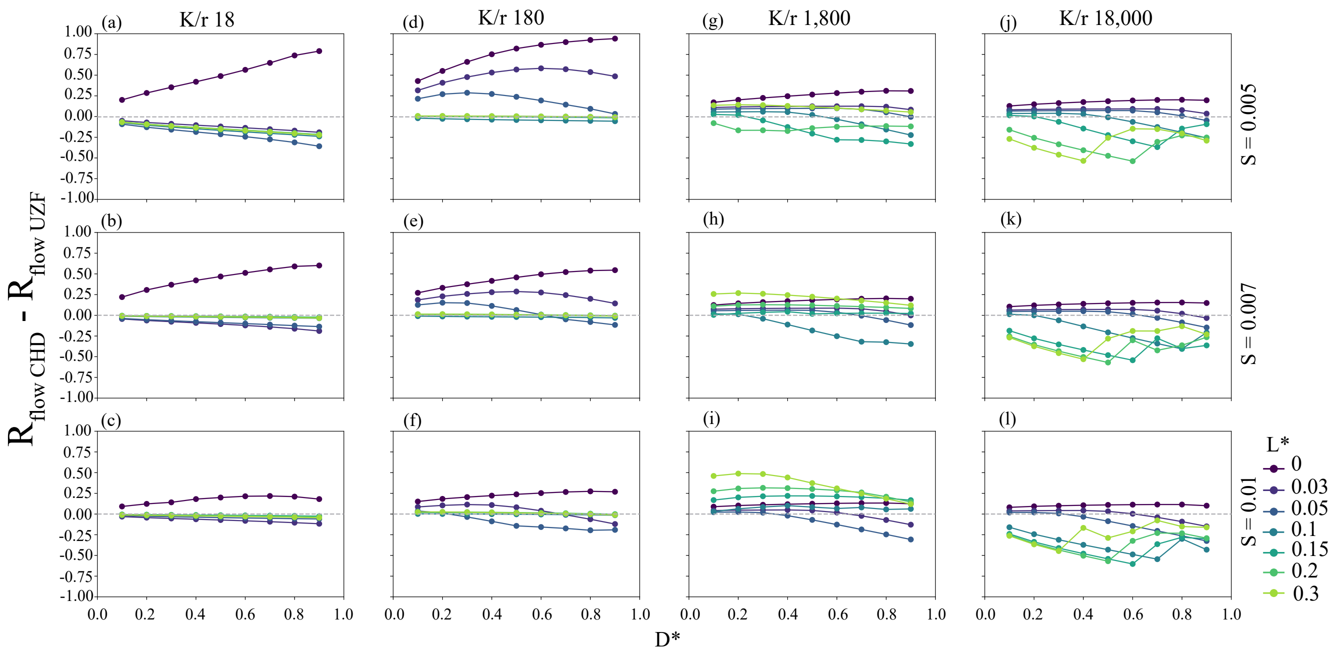

Figure S 15. R_{flow} difference between the UZF and CHD implementations for a cutoff wall ($R_{flowCHD} - R_{flowUZF}$). Columns are labeled by the K/r used for the modeling, and the rows are labeled by the topographic slope, S. Each point represents four simulations, as two simulations are needed for calculating R_{flow} for each CHD and UZF implementation.

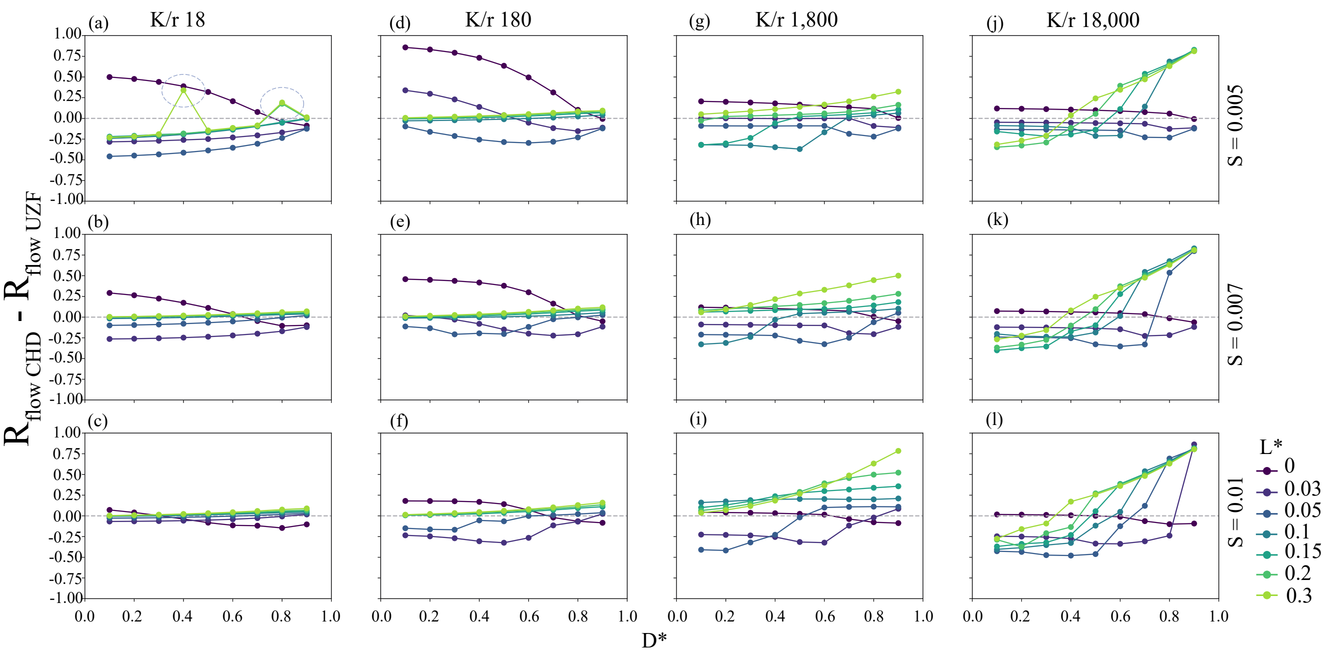

Figure S 16. R_{flow} difference between the UZF and CHD implementations for a subsurface dam. Columns are labeled by the K/r used for the modeling, and the rows are labeled by the topographic slope, S. Each point represents four simulations, as two simulations are needed for calculating R_{flow} for each CHD and UZF implementation.Dashed and circled model results were caused by numerical issues, and only happened for flat topography and very low K/r simulations.

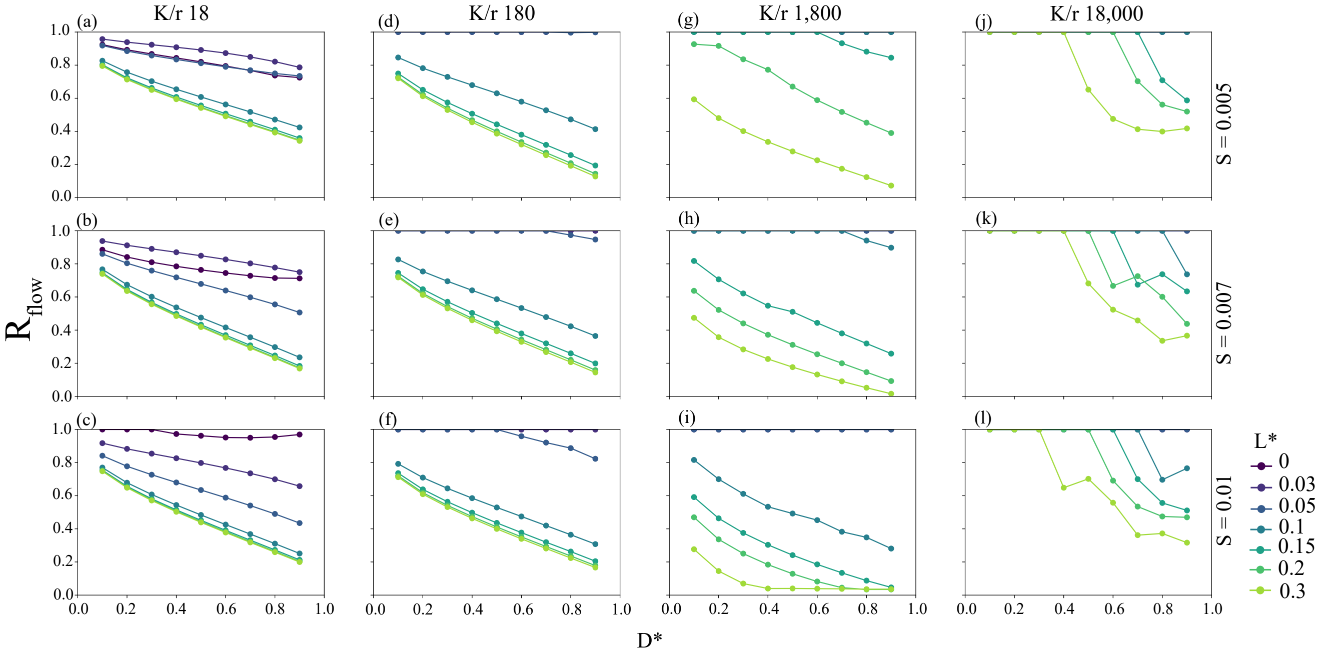

Figure S 17. Modeled inland groundwater flow ratio, R_{flow}, simulated for a cutoff wall. Columns are labeled by the K/r used for the modeling, and the rows are labeled by the topographic slope, S. Each point represents two simulations used to calculate R_{flow}, one without a barrier and one with a barrier.

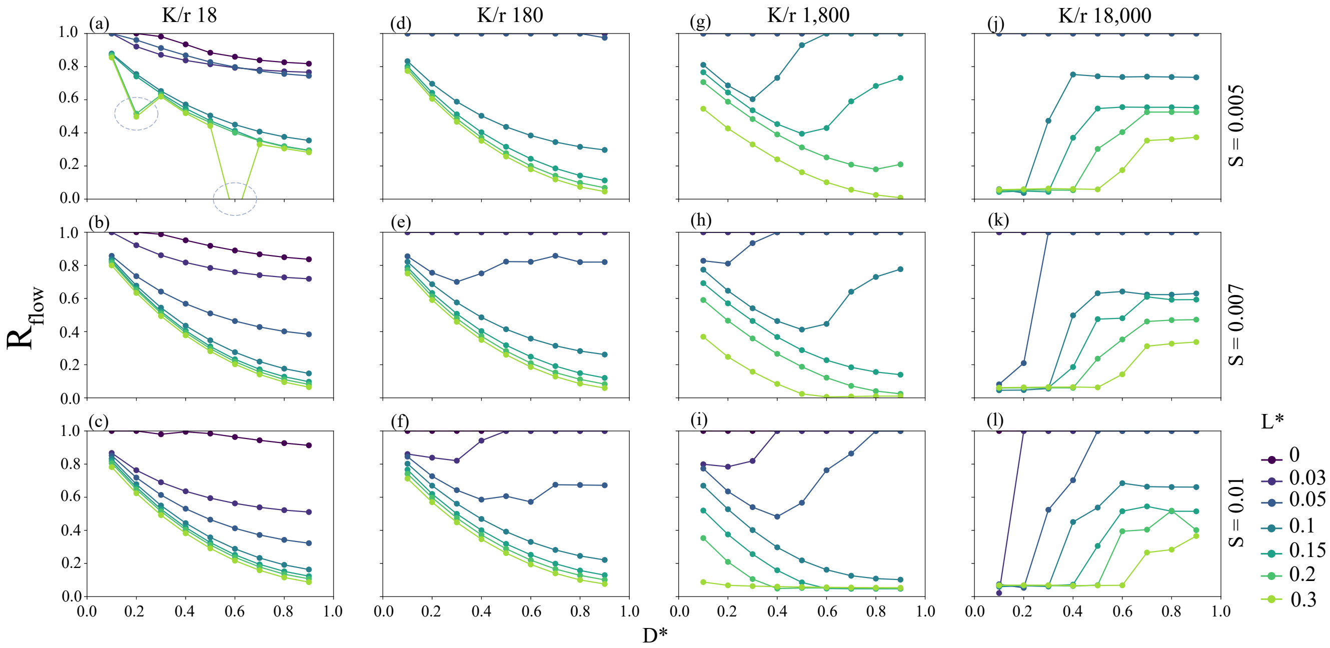

Figure S 18. Modeled inland groundwater flow ratio, R_{flow}, simulated for a subsurface dam. Columns are labeled by the K/r used for the modeling, and the rows are labeled by the topographic slope, S. Each point represents two simulations used to calculate $R_{intrusion}$, one without a barrier and one with a barrier. Models with poor convergence related to simulated instability of the saline-freshwater interface near the seafloor boundary condition are indicated with dashed circles in (a).

References

1. Befus, K., Barnard, P. L., Hoover, D. J., Finzi Hart, J. & Voss, C. I. Increasing threat of coastal groundwater hazards from sea-level rise in california. *Nat. Clim. Chang.* **10**, 946–952, DOI: 10.1038/s41558-020-0874-1 (2020).

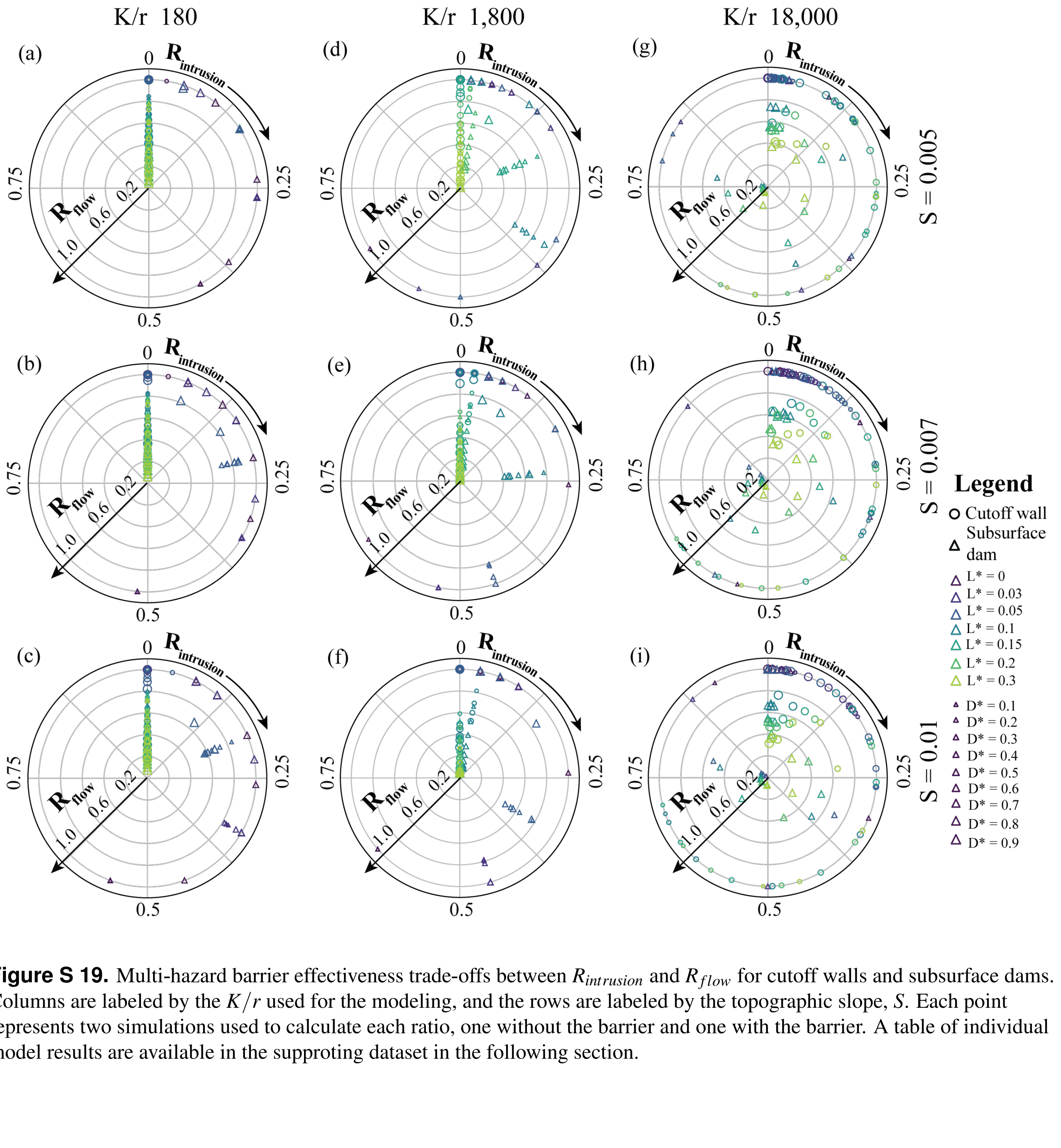

Figure S 19. Multi-hazard barrier effectiveness trade-offs between $R_{intrusion}$ and R_{flow} for cutoff walls and subsurface dams. Columns are labeled by the K/r used for the modeling, and the rows are labeled by the topographic slope, S. Each point represents two simulations used to calculate each ratio, one without the barrier and one with the barrier. A table of individual model results are available in the supproting dataset in the following section.
